# Supplementary material for: Electrochemical CO2 reduction to ethylene by ultrathin CuO nanoplate arrays
Source: Nat Commun. 2022 Apr 6;13:1877. doi: 10.1038/s41467-022-29428-9 (PMC8986799; doi:10.1038/s41467-022-29428-9)
Supplement: Supplementary file 1 — Supplementary Information [file 41467_2022_29428_MOESM1_ESM.pdf]

Supplementary Information

Electrochemical CO_2 reduction to ethylene by ultrathin CuO nanoplate arrays

Wei Liu[1], Pengbo Zhai[2], Aowen Li[3,4], Bo Wei[1], Kunpeng Si[1], Yi Wei[5,6], Xingguo Wang[1], Guangda Zhu[7], Qian Chen[1], Xiaokang Gu[1], Ruifeng Zhang[1], Wu Zhou[3,4,8] and Yongji Gong[1,9]*

[1] *School of Materials Science and Engineering, Beihang University, Beijing 100191, China.*
[2] *College of Physics, Qingdao University, Qingdao 266071, China.*
[3] *School of Physical Sciences, University of Chinese Academy of Sciences, Beijing 100049, China.*
[4] *CAS Key Laboratory of Vacuum Physics, University of Chinese Academy of Sciences, Beijing 100049, China.*
[5] *State Key Laboratory of Organic-Inorganic Composites, Beijing University of Chemical Technology, Beijing 100029, China.*
[6] *Beijing Key Laboratory of Electrochemical Process and Technology for Materials, Beijing University of Chemical Technology, Beijing 100029, China.*
[7] *Beijing National Laboratory for Molecular Sciences, Laboratory of Polymer Physics and Chemistry, Institute of Chemistry, Chinese Academy of Sciences, Beijing 100190, China.*
[8] *CAS Center for Excellence in Topological Quantum Computation, University of Chinese Academy of Sciences, Beijing 100049, China.*
[9] *Center for Micro-Nano Innovation of Beihang University, Beijing 100191, China.*

E-mail address: yongjigong@buaa.edu.cn

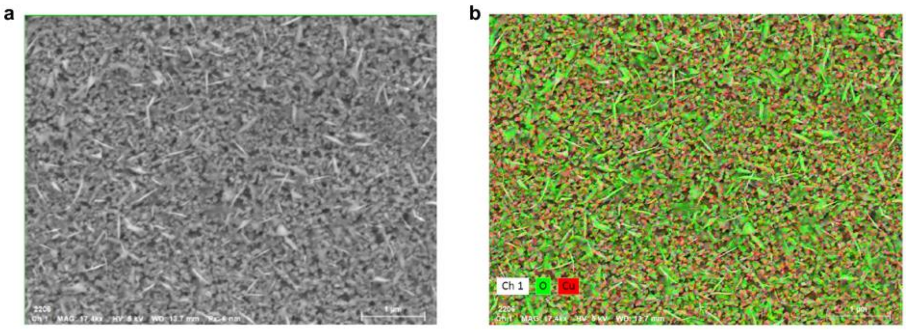

Supplementary Fig. 1 SEM image and EDS maps of Cu foil after 15 min of electrolysis. The acicular nanoplates are uniformly distributed on the Cu surface. In the EDS mapping, the oxygen element (in green color) is enriched in the nanoplates region, which suggests the composition of nanoplates is CuO.

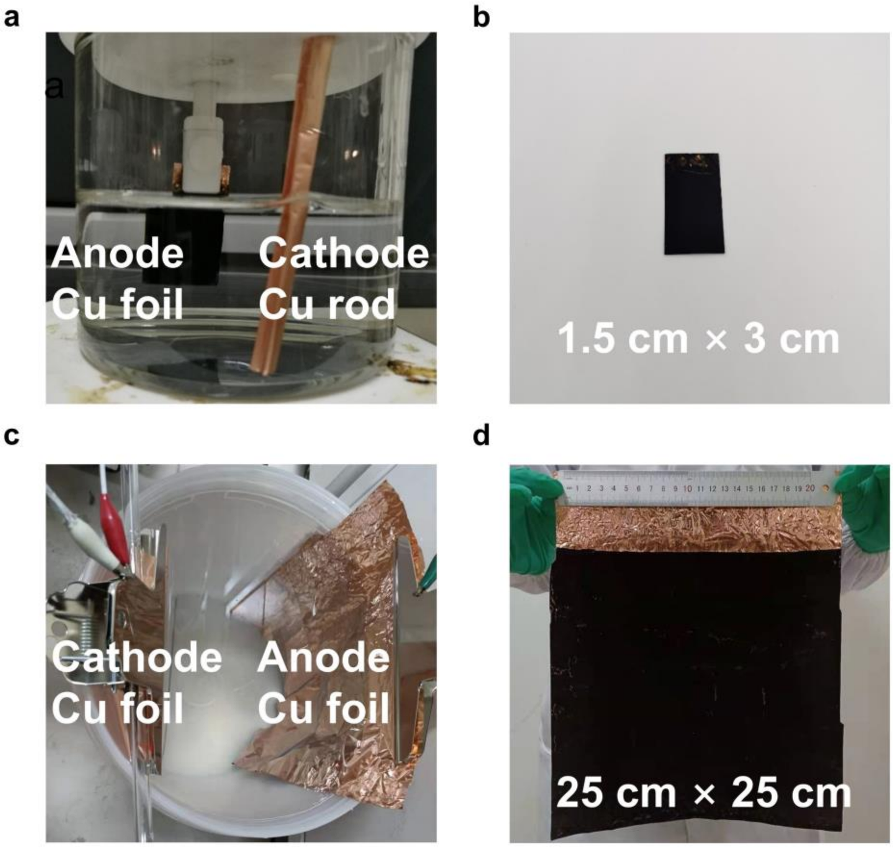

Supplementary Fig. 2 Optical images of electrolysis devices and CuO-NPs on Cu foils in different sizes. In the electrolysis devices, Cu foils were used as anode and cathode (or Cu rod rolled by Cu foil). The anodic current densities were set as 0.26 mA cm^{-2} for both sizes' Cu foils. CuO-NPs on two sizes' Cu foils could be successfully synthesized.

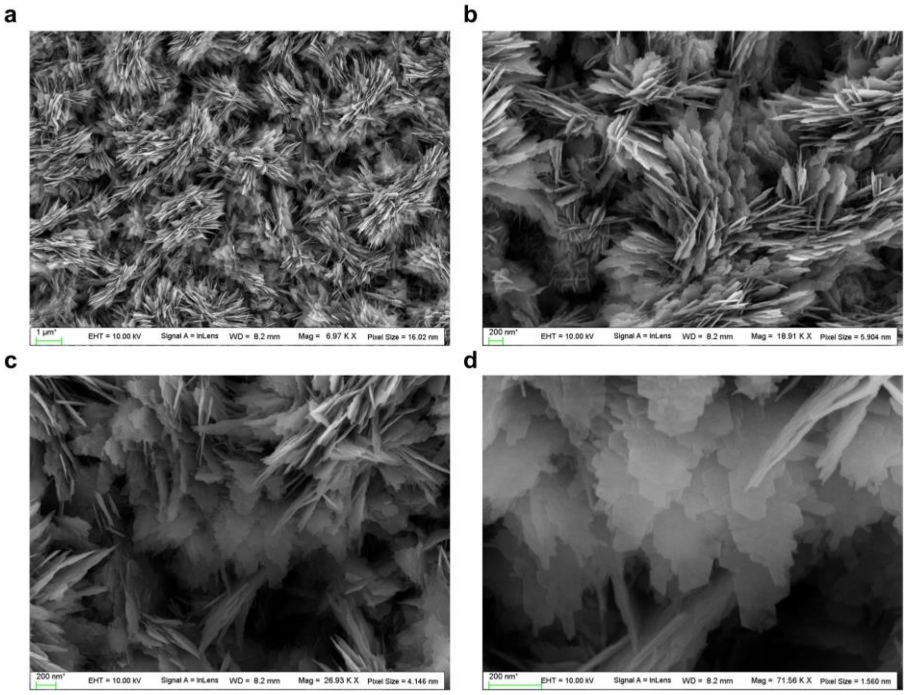

Supplementary Fig. 3 SEM images of CuO-NPs on 25 × 25 cm^2 Cu foil at different magnification. CuO-NPs could be synthesized on different sized Cu foils.

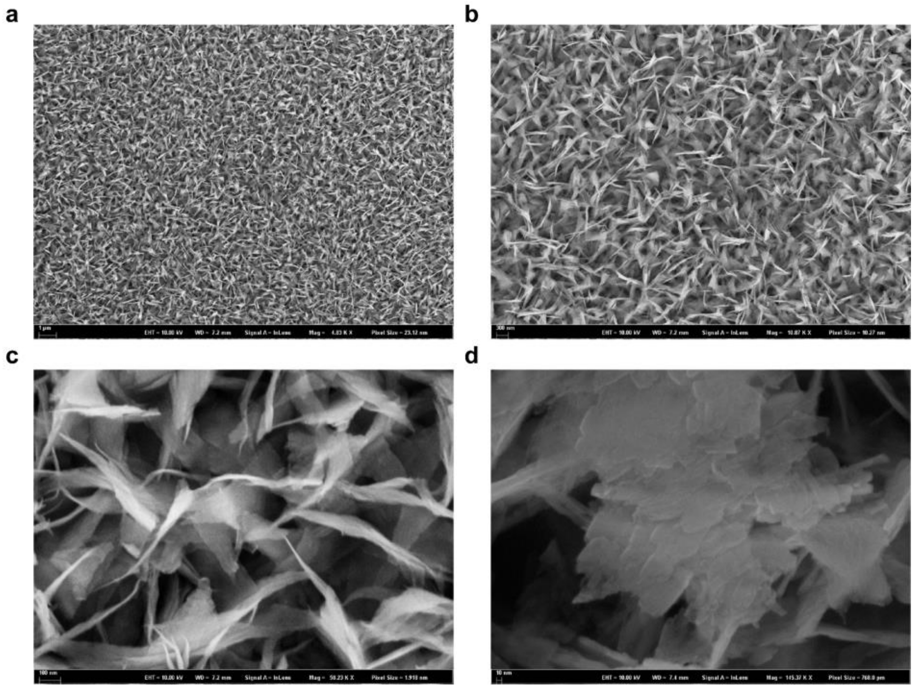

Supplementary Fig. 4 SEM images of CuO-NPs at different magnification. The low-magnification SEM images show the CuO flakes are vertically arranged and densely stacked on the Cu surface. The high-magnification SEM image indicates that the CuO flakes are about 10 nm in thickness and composed of tiny polygonal CuO nanosheets.

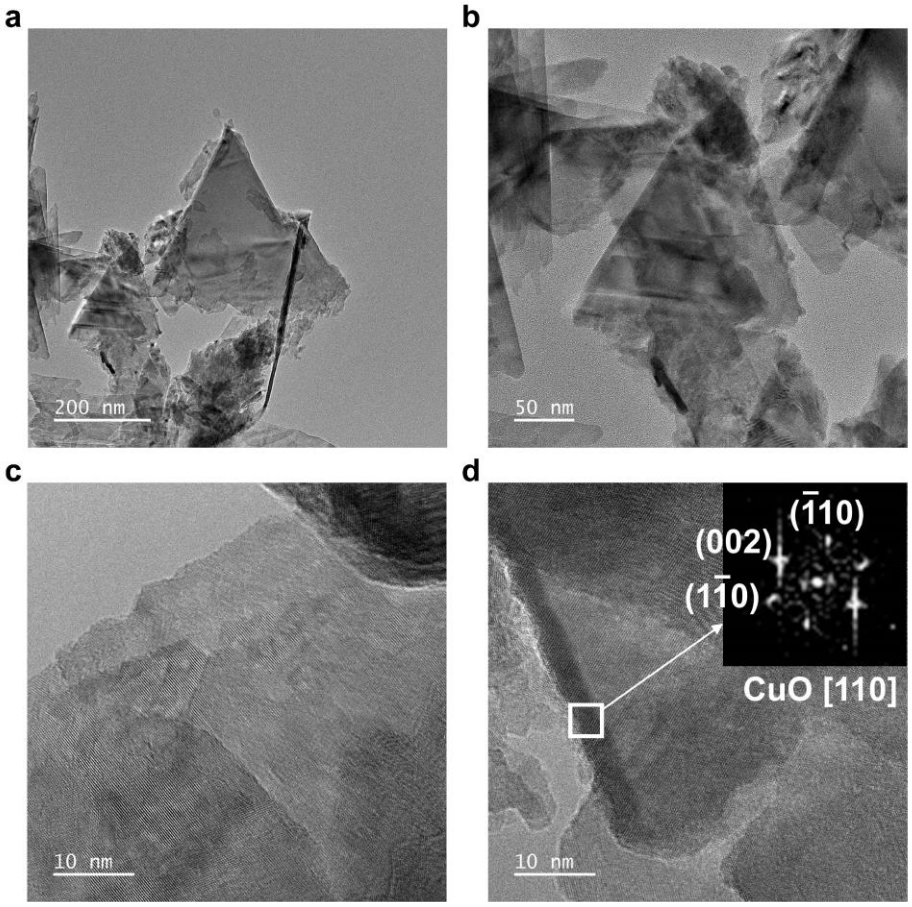

Supplementary Fig. 5 TEM images of CuO-NPs at different magnifications. The TEM images of CuO-NPs indicate that CuO-NPs consist of polygonal CuO nanosheets. The high-resolution transmission electron microscopy (HRTEM) image presents good crystallization of CuO-NPs. The fast Fourier transform (FFT) pattern of the selected area corresponds to the CuO [110] zone axis.

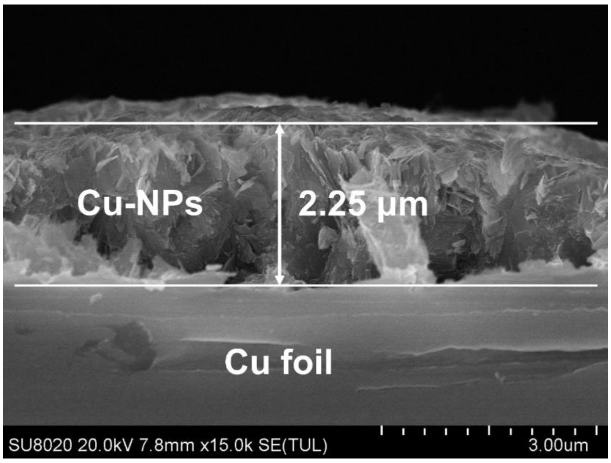

Supplementary Fig. 6 Cross-sectional SEM image of CuO-NPs. The cross-sectional SEM image indicates the thickness of the CuO-NPs layer is about 2.25 μm.

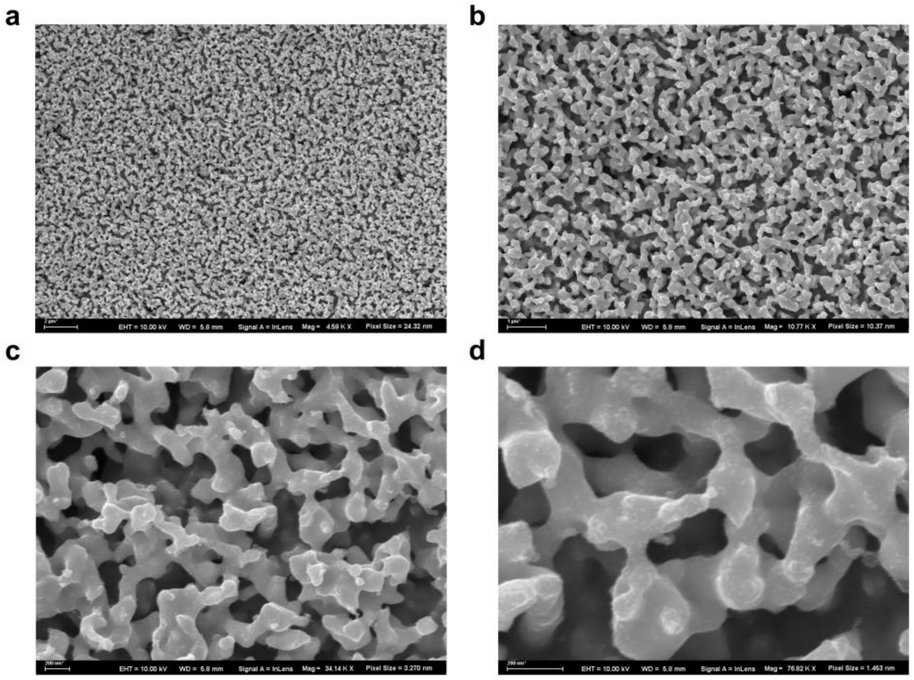

Supplementary Fig. 7 SEM images of R-CuO-NPs at different magnifications. The SEM images of R-CuO-NPs exhibit three-dimensional porous nanostructure after the thermal reduction in Ar/H_2.

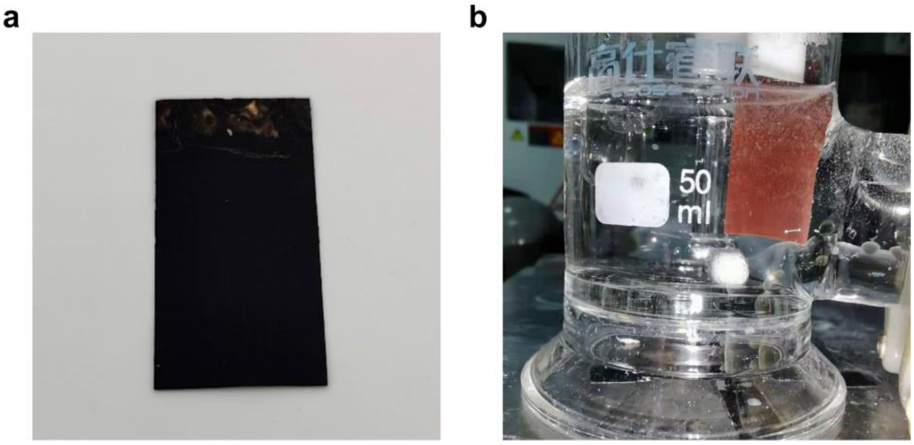

Supplementary Fig. 8 Optical images of CuO-NPs and DVL-Cu. The black CuO-NPs foil quickly turned to rufous when applied negative bias. Fig. **b** was taken after 10 min electrolysis at −0.8 V.

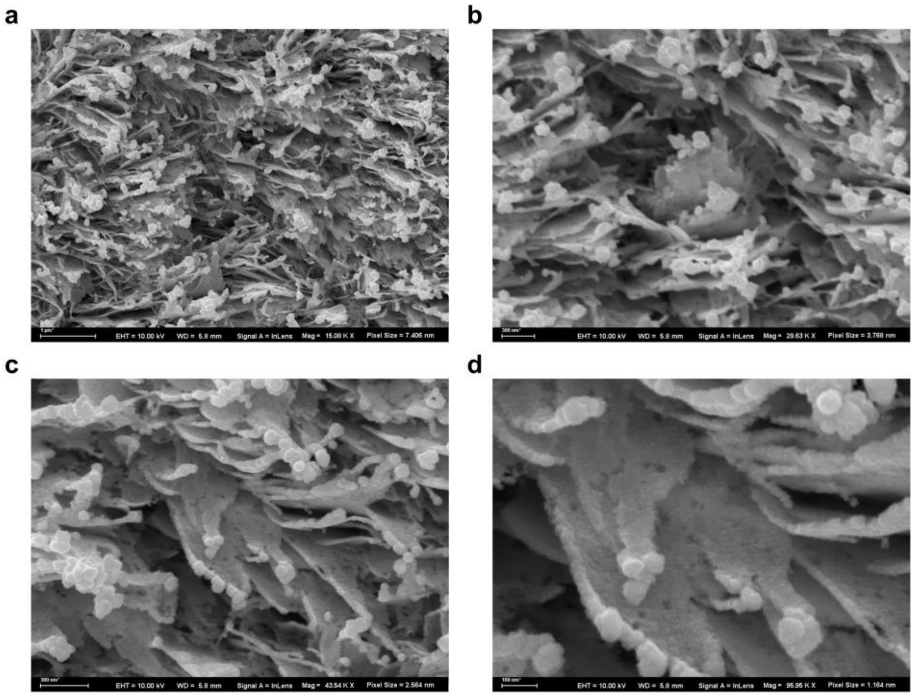

Supplementary Fig. 9 SEM images of DVL-Cu at different magnifications. The SEM images of DVL-Cu indicate that the vertically arranged and densely stacked laminated nanostructures are retained after electrochemical reduction. However, the flakes are no longer composed of CuO polygonal nanosheets but are composed of nanoparticles.

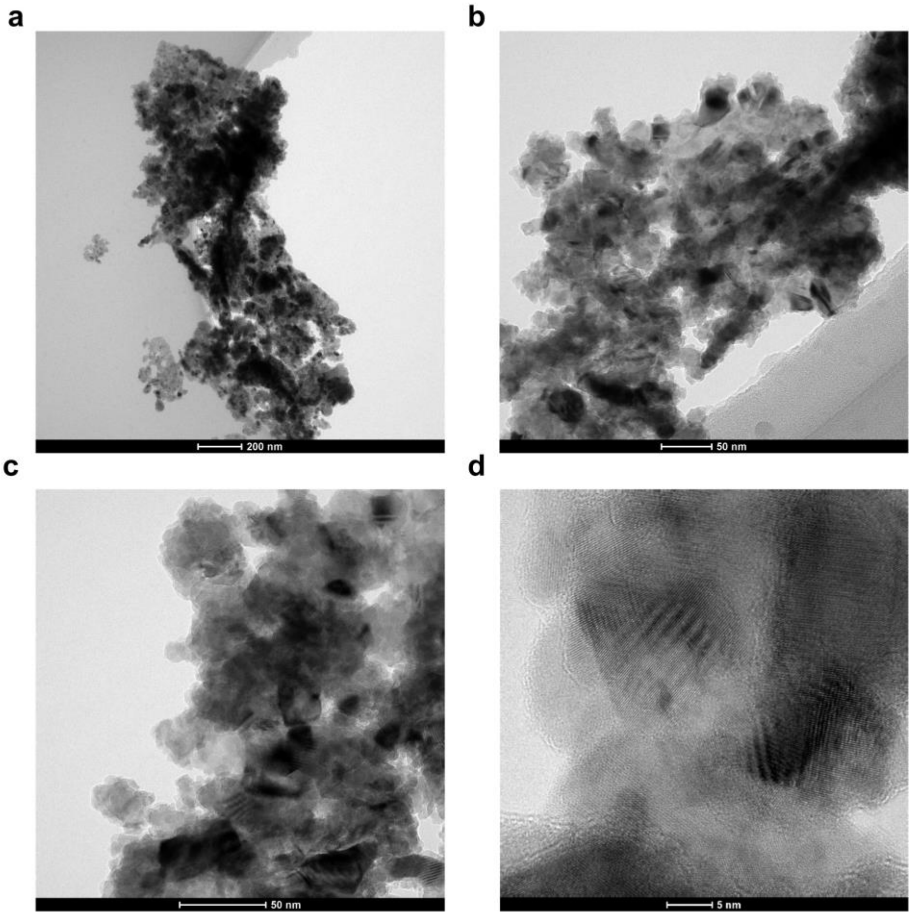

Supplementary Fig. 10 TEM images of DVL-Cu at different magnifications. DVL-Cu are composed of nanoparticles.

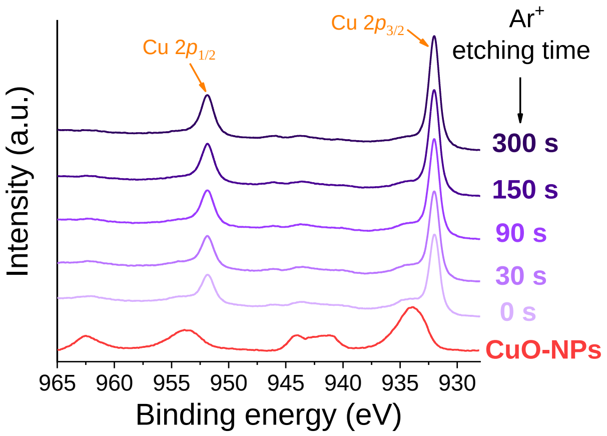

Supplementary Fig. 11 Cu XPS 2*p* spectra of CuO-NPs and DVL-Cu (after 1 h cathodic reduction) with respect to different Ar^+ etching times. The typical satellite peaks of CuO-NPs indicate that the Cu is successfully oxidized to CuO after anodic oxidation. The DVL-Cu sample taken after CO_2RR shows consistent Cu^0/Cu^+ 2*p* peaks. The peak intensities increase as the etching time extended, probably due to the increase content of the Cu element in the deeper subsurface.

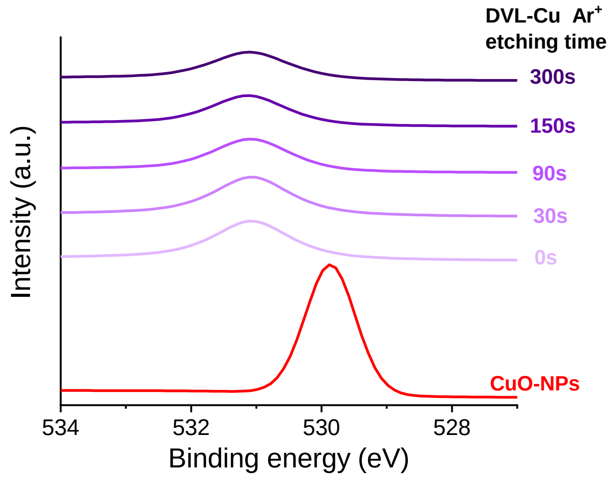

Supplementary Fig. 12 O XPS 1*s* spectra of CuO-NPs and DVL-Cu (after 1 h cathodic reduction) with respect to different Ar^{+} etching times. The peaks located at 529.9 eV (CuO-NPs) and 531.6 eV (DVL-Cu) could be ascribed to CuO and Cu_2O, respectively. The peak intensities of O decrease as the etching time extended, probably due to the increase of the Cu element in the deeper subsurface.

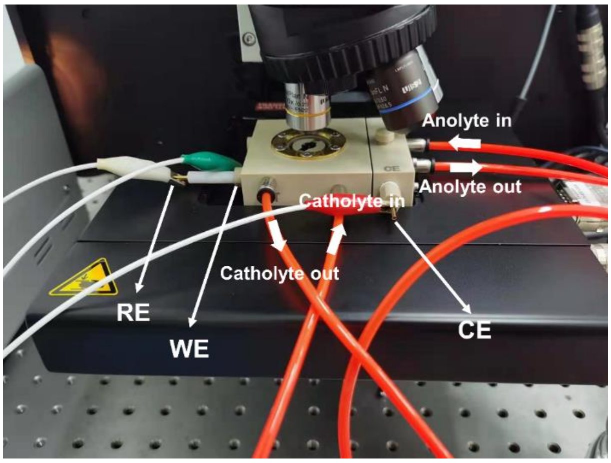

Supplementary Fig. 13 Optical image of the *in situ* Raman electrolyzer for CO_2RR. The catholyte and anolyte were CO_2-saturated 0.5 M KCl solution. A two-channel peristaltic pump was employed to circulate the electrolyte during the test.

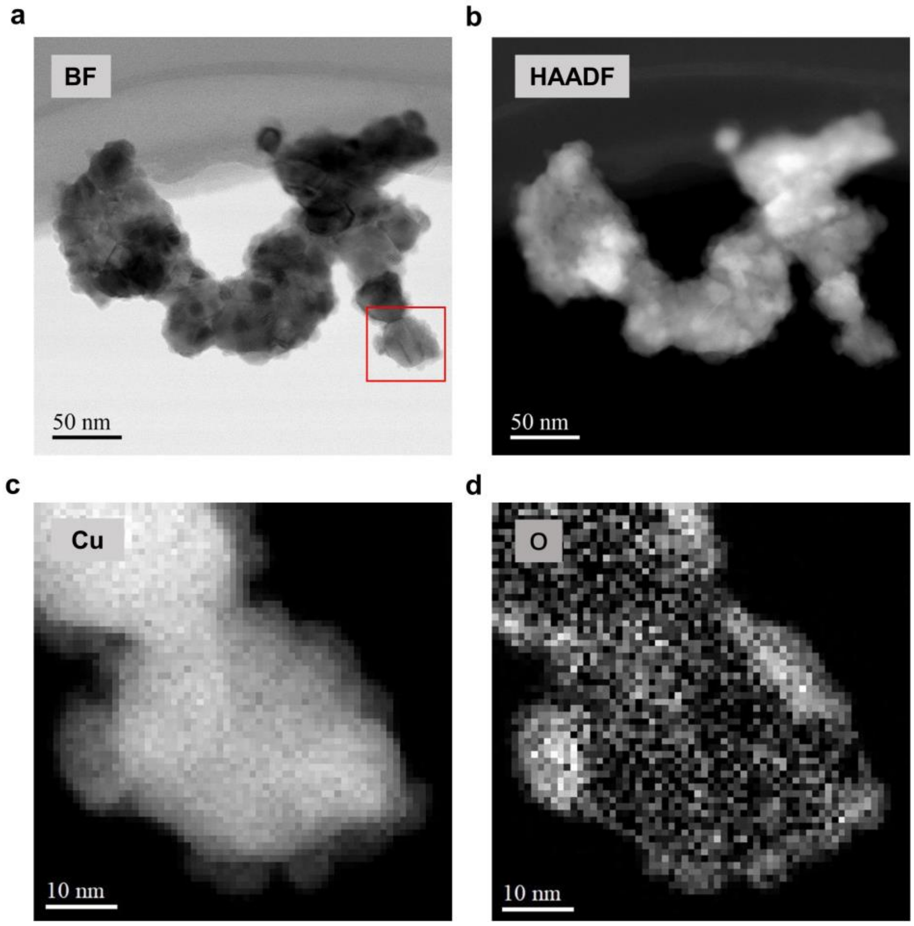

Supplementary Fig. 14 STEM imaging and EELS elemental mapping of DVL-Cu. The area in Fig. 3 is circled in (**a**). (**c**) and (**d**) are the Cu and O EELS elemental maps of the area in Fig. 3**a**, respectively, implying that the small nanoparticles around are Cu_xO rather than metallic Cu.

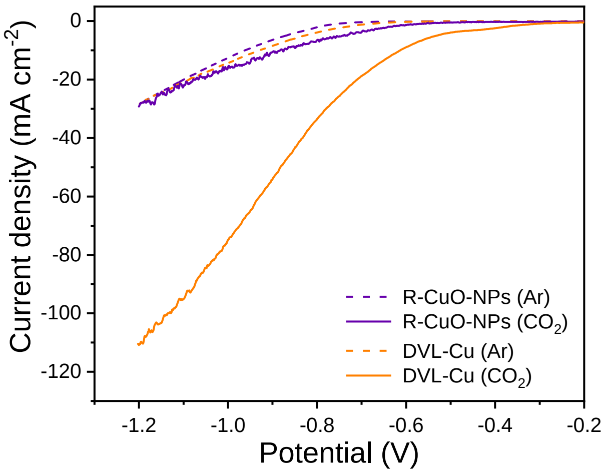

Supplementary Fig. 15 LSV curves of DVL-Cu and R-CuO-NPs in Ar and CO_2 saturated KCl electrolyte. The current density of DVL-Cu (CO_2) is larger than that of R-CuO-NPs catalyst (CO_2), indicating that DVL-Cu possesses higher intrinsic CO_2RR activity.

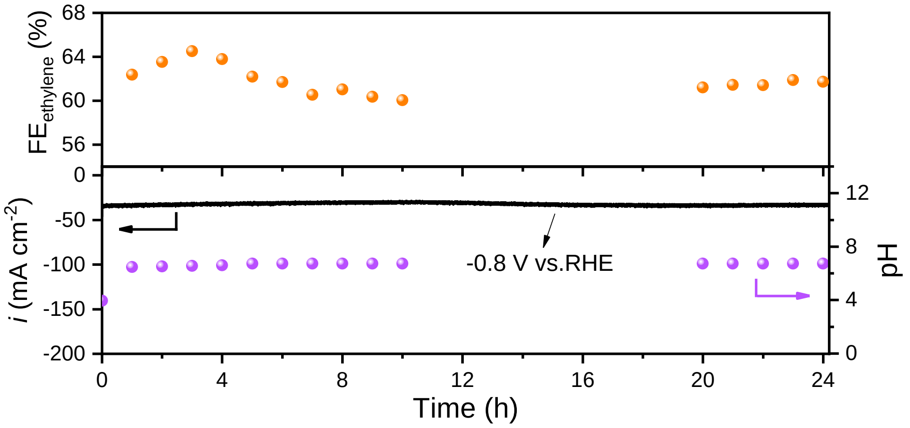

Supplementary Fig. 16 pH and ethylene FE during the long-term test in H-cell. pH value raised rapidly from 3.90 to 6.52 in the initial 1 h. Then pH value retained around 6.7 for the following test, a neutral catholyte environment. Meanwhile, the FE of C_2H_4 remained stable in this pH range, indicating good catalytic performance could be achieved in the neutral catholyte.

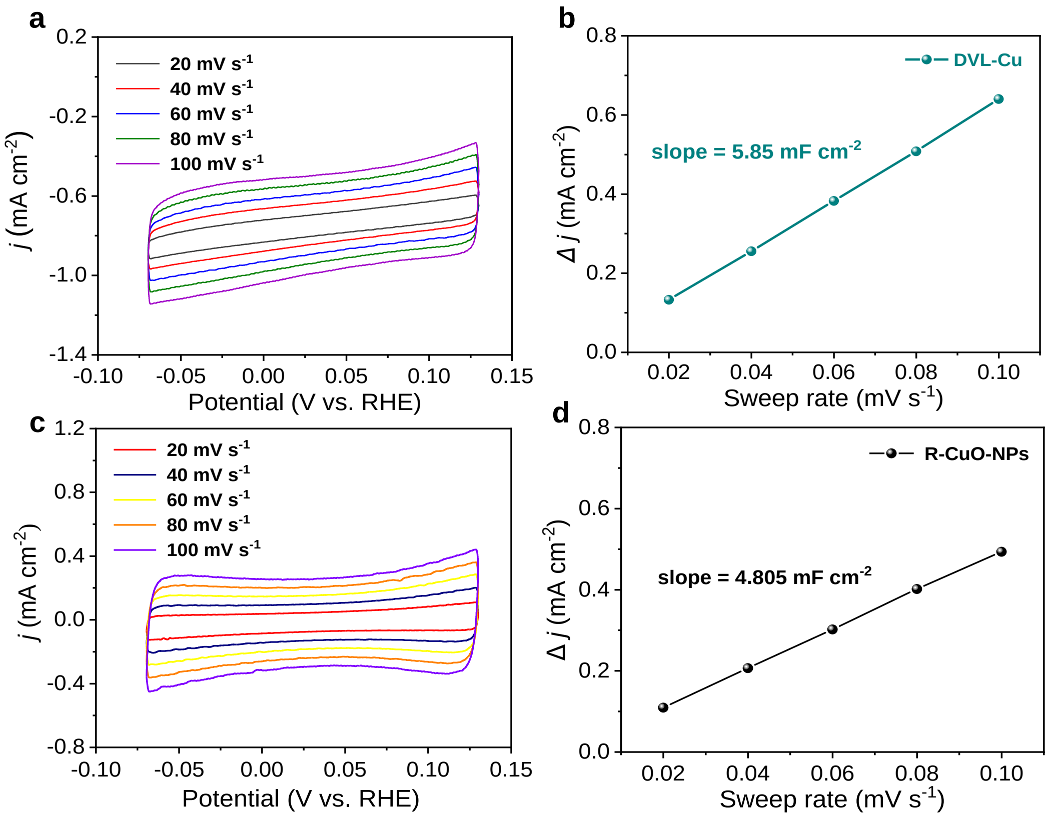

Supplementary Fig. 17 ECSA measurements of DVL-Cu and R-CuO-NPs catalysts. a, **b** The cyclic voltammetry (**a**) and double layer capacitance (**b**) curves of the DVL-Cu catalyst. c,d, The cyclic voltammetry (**c**) and double layer capacitance (**d**) curves of R-CuO-NPs. The reference double-layer capacitance of the polycrystalline Cu[1] is 29 μF cm^{-2}.

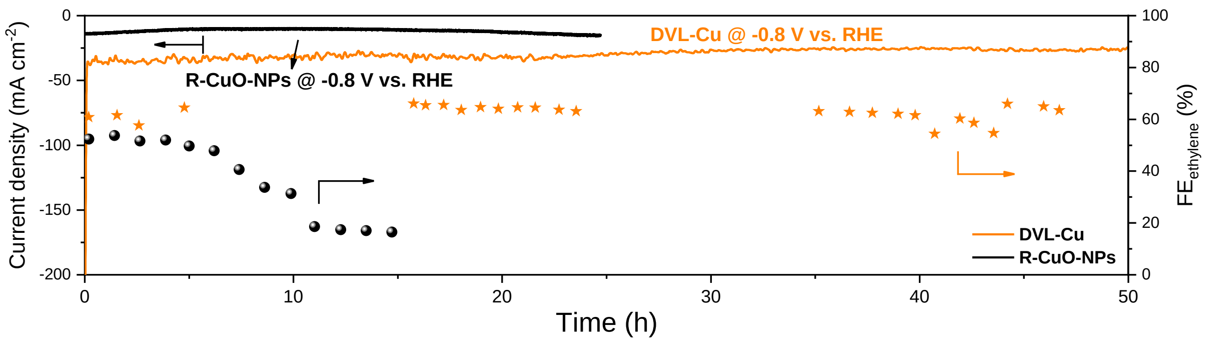

Supplementary Fig. 18 Stability test of DVL-Cu and R-CuO-NPs at constantly applied potentials. The slight fluctuation in FEs was caused by the temperature difference between day and night.

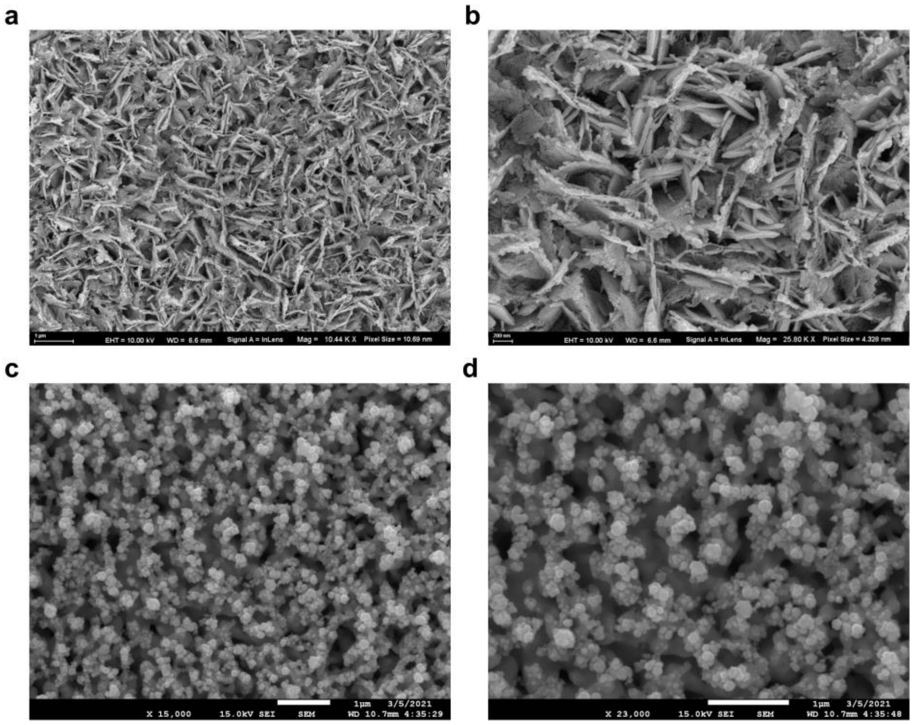

Supplementary Fig. 19 SEM images of DVL-Cu and R-CuO-NPs taken after 2 h CO_2RR. **a, b** SEM image of the 2 h post-electrolysis DVL-Cu at different magnifications. **c, d** SEM image of the 2 h post-electrolysis R-CuO-NPs at different magnifications. Small Cu nanoparticles agglomerate on the surface of R-CuO-NPs.

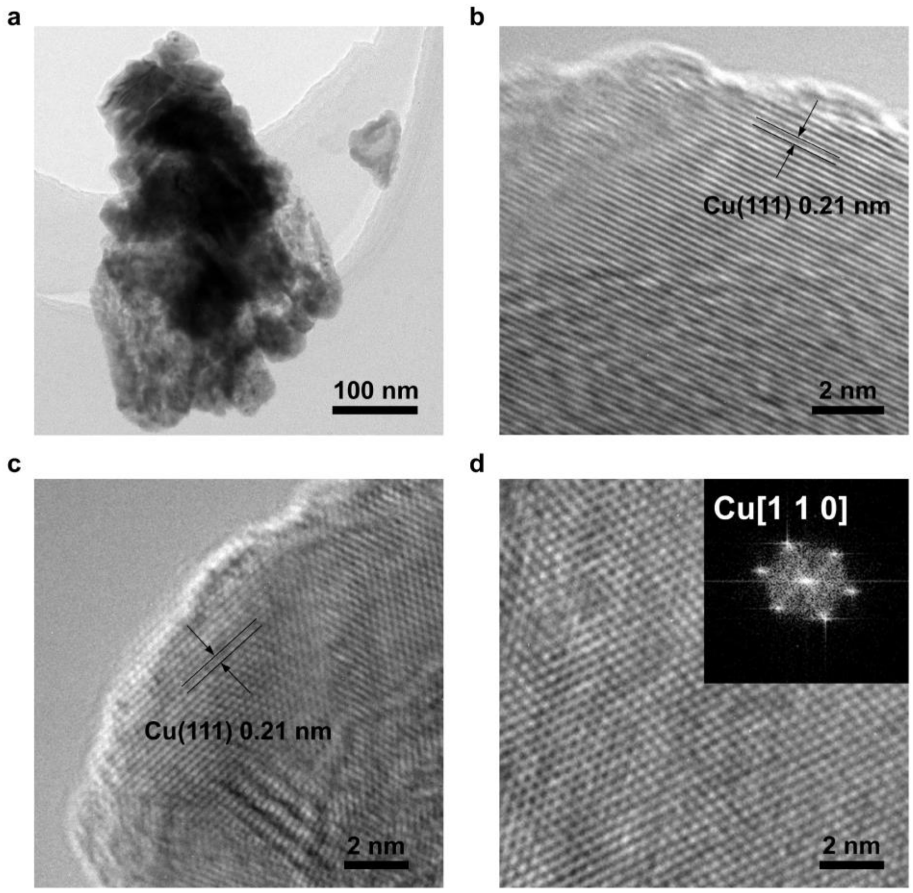

Supplementary Fig. 20 TEM images of R-CuO-NPs taken after 2 h CO_2RR. The FFT patterns of the HRTEM image taken at the edge (**c**) or center (**d**) in the spent R-CuO-NPs are consistent to [110] zone axis (**d**). Unlike the DVL-Cu catalyst, no Cu_2O species is found in R-CuO-NPs.

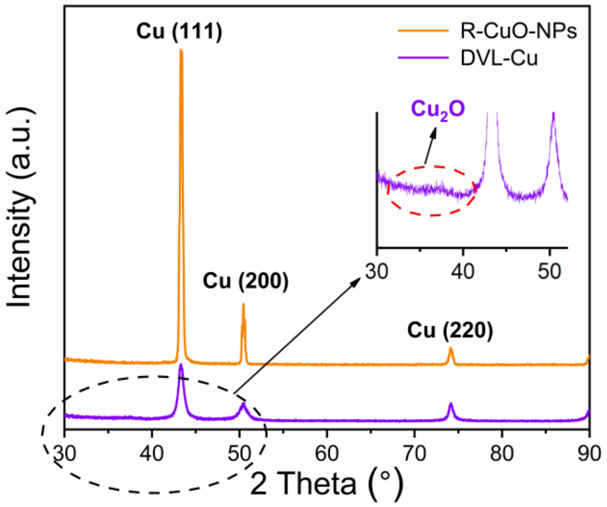

Supplementary Fig. 21 GI-XRD profiles of R-CuO-NPs and DVL-Cu taken after 1 h CO_2RR. There are no Cu_2O species in R-CuO-NPs. The peaks located at 43.2°, 50.5°, and 74.1° are corresponding to Cu(111), (200), and (220), respectively. The Cu_2O characteristic peak still exists in DVL-Cu after 1 h CO_2RR, while it is absent in R-CuO-NPs.

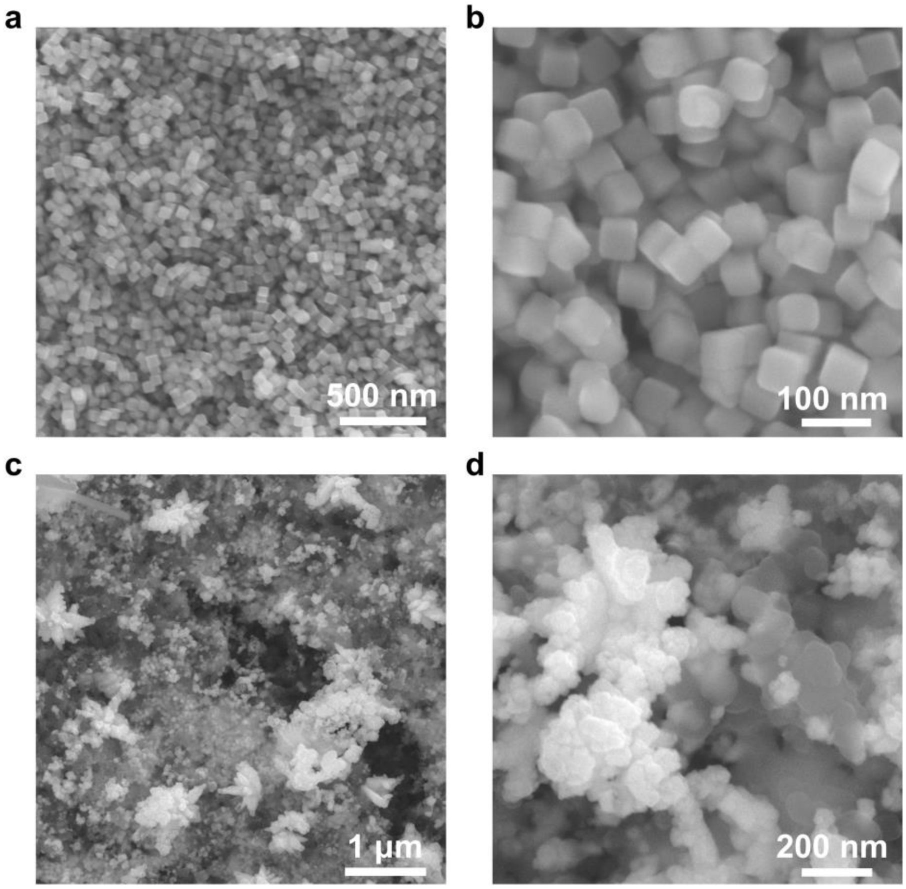

Supplementary Fig. 22 SEM images of Cu_2O nanocubes. a, b SEM images of Cu_2O nanocubes at different magnifications. **c, d** SEM images of Cu_2O nanocubes after 1 h CO_2RR at different magnifications.

Supplementary Note 1: Electrolyte effect.

In order to reveal the underlying mechanism of the electrolyte, we selected $KHCO_3$ (buffer electrolyte) and K_2SO_4 (non-buffer electrolyte without Cl^-) for analysis. Firstly, the catalytic performance of DVL-Cu in these electrolytes was measured. Total current density plots with saturated CO_2 are shown in Fig. 6a. Current densities obtained from these electrolytes are very similar. The current density in K_2SO_4 is slightly higher than that in $KHCO_3$ and KCl. Besides, FEs of products may be more informative. The peak ethylene FE in $KHCO_3$ and K_2SO_4 is 66.4% (−0.7V) and 72.8% (−0.8V), respectively (Figs. 6b and c). Both ethylene FEs decline when the potential goes further negative, with increased hydrogen FEs. The methane production in K_2SO_4 is nearly negligible. In contrast, methane FE reaches 17.8% in $KHCO_3$ at −1.1V. By observing the overall product distribution, we can find that the ethylene production in $KHCO_3$ is not satisfied. The ethylene FEs exceed 60% only in the limited potential range and the methane production is not well suppressed at higher overpotentials. Ethylene FEs in K_2SO_4 at moderate overpotentials are similar to KCl, but hydrogen production dominates at higher overpotentials.

The performance difference can be explained by the following reasons. $KHCO_3$ is a buffering electrolyte and the local generated OH^- during CO_2RR could be neutralized by HCO_3^-, leading to lower local pH than the non-buffering electrolyte. We conclude that the lower local pH in $KHCO_3$ results in inferior ethylene production because lower pH regions could be favorable to hydrogen evolution and methane production, both needing H^+. Then, higher local pH generated in non-buffering electrolytes would suppress methane production in all potential

ranges and hydrogen evolution at lower overpotentials. Comparing the catalytic performance in KCl and K_2SO_4, it could be concluded that the existence of Cl^- could suppress hydrogen evolution even at high overpotentials, which is considered the result of the Cl^- specific adsorption effect. The strongly adsorbed Cl^- could facilitated the electron transfer from the electrode to CO_2 and suppress the adsorption of protons, leading to a higher hydrogen evolution overpotential.

Subsequently, long-term performances in different electrolytes were tested to explore the relationship between catalyst stability and electrolyte (Fig. 6d). Interestingly, DVL-Cu delivered ~50h stable current density and ethylene FE in K_2SO_4 under −0.8V. In comparison, it only preserved stable performance for less than 10h in $KHCO_3$ under the same condition. SEM and TEM images of the post-CO_2RR sample in K_2SO_4 (Fig. 7) displays equally morphology and Cu(I)/Cu(0) interfaces with that gained from the KCl sample, indicating that high local pH plays a critical role for the prolonged stability. Detailed post-electrolysis characterizations were performed to unravel the underlying mechanism of stability difference for the sample obtained from $KHCO_3$ and KCl. As shown in Figs. 8c and d, a more severe Cu agglomeration on the top of nanoplates can be seen from the $KHCO_3$ sample. AC-TEM and EELS mapping were carried out to distinguish both samples' nanostructure and Cu(I) species distribution. Both samples are nanoplates composed of tiny nanoparticles with almost the same morphology (Figs. 9a, 10a). EELS mapping of the $KHCO_3$ sample (Figs. 9b-d) reveals that Cu(I) species agglomerate on the top of nanoplates while these species distribute uniformly in the KCl sample (Figs. 10d-f). AES depth profile analyses (Fig. 11) further verify the Cu(I)

species distribution differences, where the Cu(I) content in KCl sample is more and more uniform along with depth compred to the $KHCO_3$ sample. Apparently, non-buffering electrolytes could stable the DVL-Cu catalyst by protecting its Cu(I)/Cu(0) interfaces during electrolysis. In view of the fact that the K_{sp} of CuOH is relatively low ($1.0*10^{-14}$), high local pH could significantly slow down the dissolution of Cu(I) species. Hence, high local pH would suppress the dissolution/redeposition process of Cu(I) species in non-buffering electrolytes, which preserve the Cu(I)/Cu(0) interfaces during CO_2RR process.

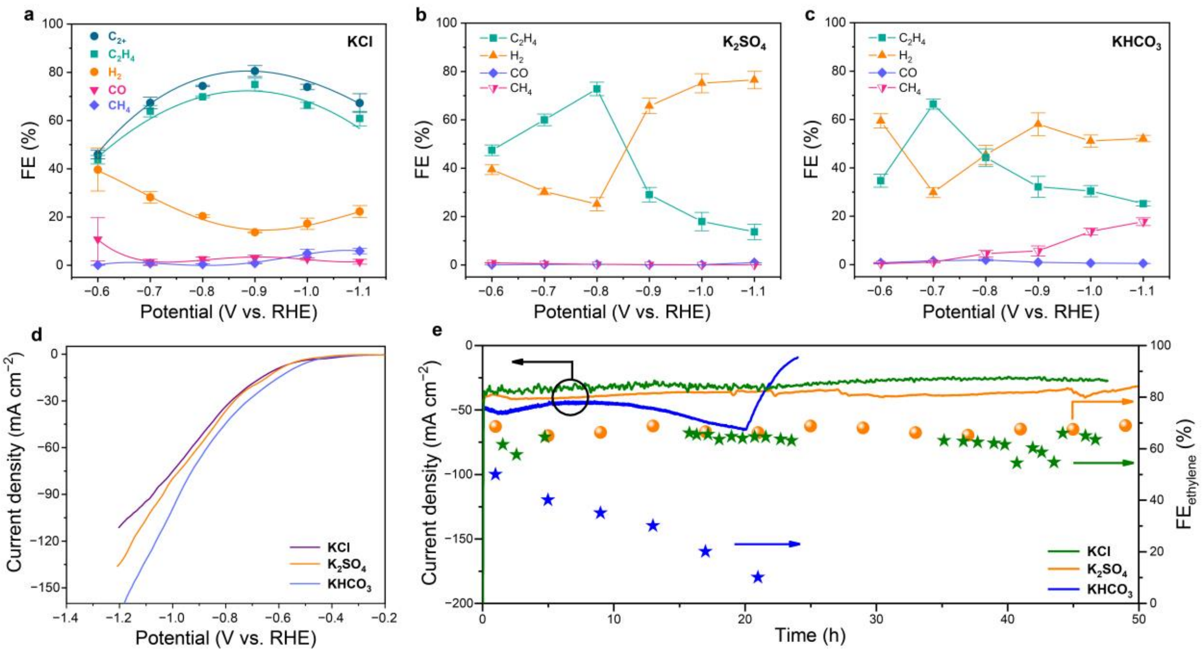

Supplementary Fig. 23 Catalytic performance of DVL-Cu in different electrolytes. a FEs of DVL-Cu in 0.5 M KCl. **b** FEs of DVL-Cu in 0.25 M K_2SO_4. **c** FEs of DVL-Cu in 0.5 M $KHCO_3$. **d** LSV curves of DVL-Cu in 0.5 M KCl, 0.5 M $KHCO_3$ and 0.25 M K_2SO_4 during CO_2RR. **e** Stability test of DVL-Cu in 0.5 M KCl, 0.5 M $KHCO_3$ and 0.25 M K_2SO_4 at −0.8V. Error bars represent the standard deviation of three independent measurements.

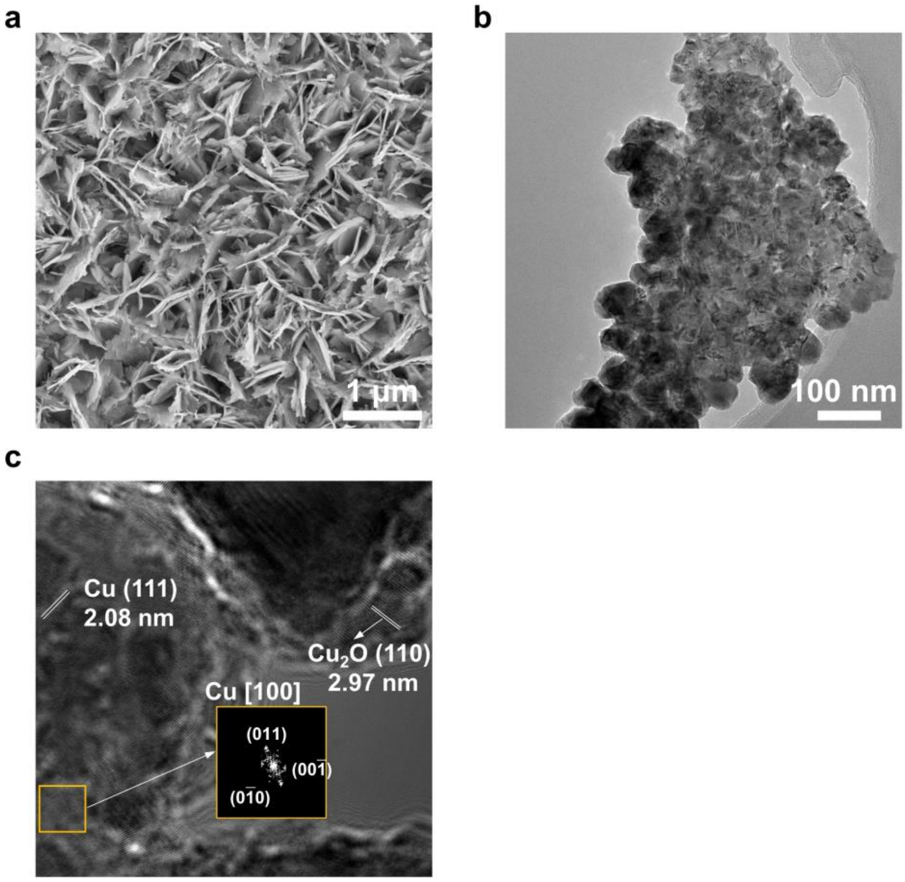

Supplementary Fig. 24 Characterizations of DVL-Cu after 50 h electrolysis in K_2SO_4. a SEM image of the 50 h post-electrolysis DVL-Cu in K_2SO_4. **b, c** TEM images of the 50 h post-electrolysis DVL-Cu in K_2SO_4 at different magnifications.

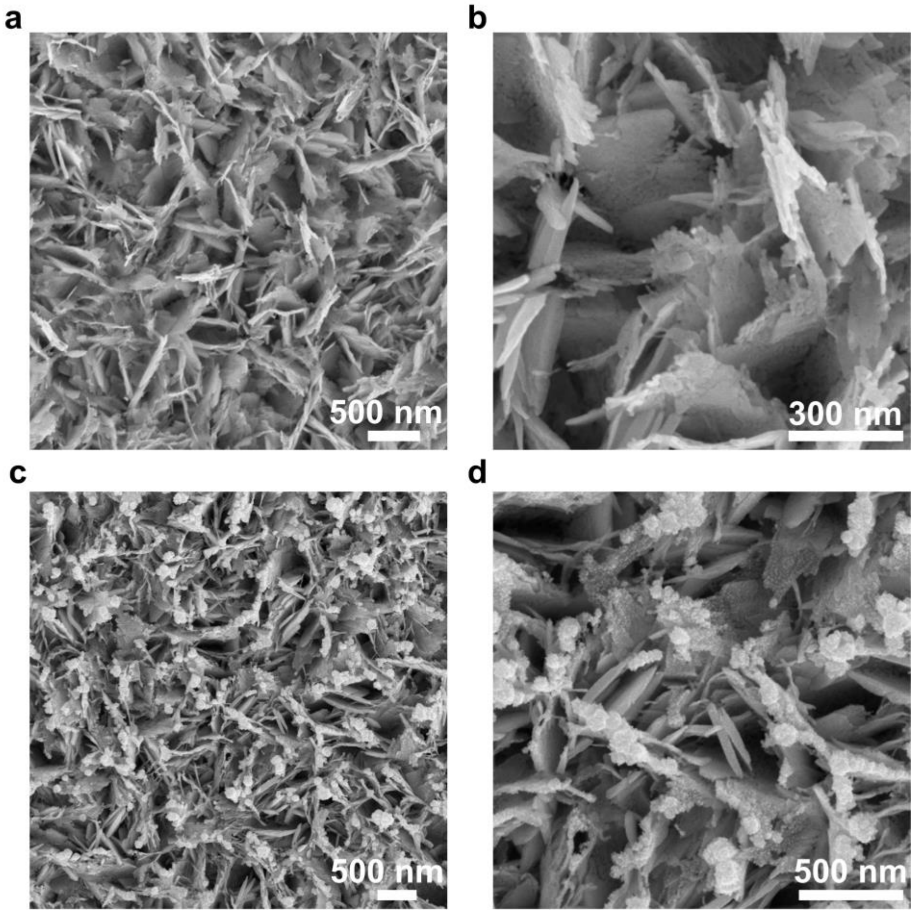

Supplementary Fig. 25 Characterizations of DVL-Cu after 50 h electrolysis in KCl. a, b SEM images of the 50 h post-electrolysis DVL-Cu in KCl at different magnifications. **c, d** SEM images of the 20 h post-electrolysis DVL-Cu in $KHCO_3$ at different magnifications.

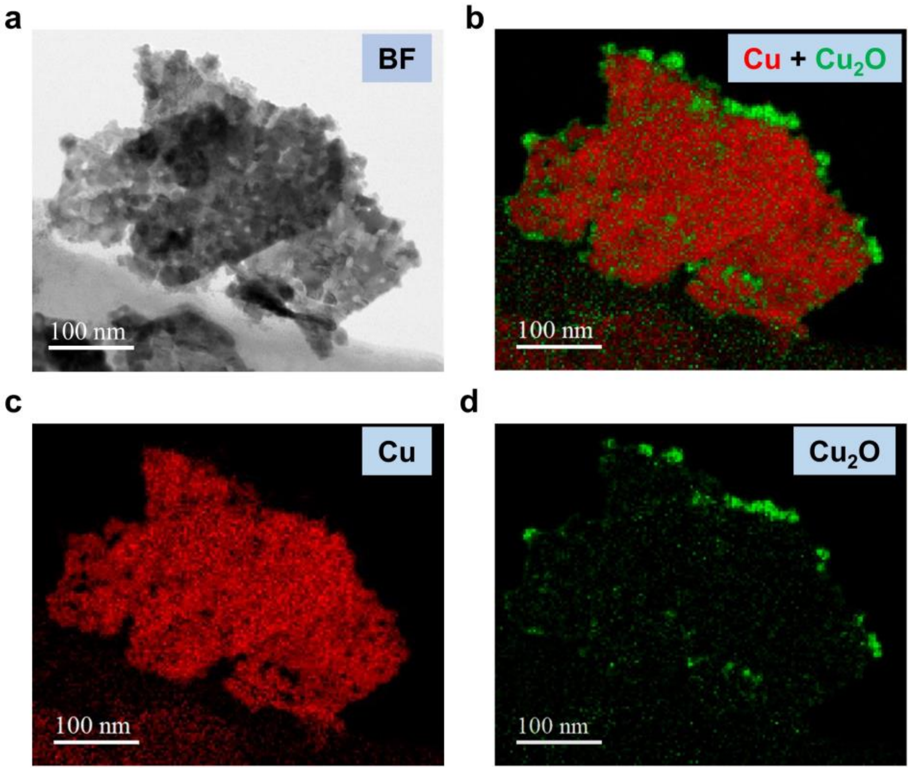

Supplementary Fig. 26 Characterizations of DVL-Cu after 50 h electrolysis in $KHCO_3$. a STEM bright-field image of the 20 h post-electrolysis DVL-Cu in $KHCO_3$. **b-d** EELS maps of Cu, Cu_2O and their overlay in the 20 h post-electrolysis DVL-Cu in $KHCO_3$.

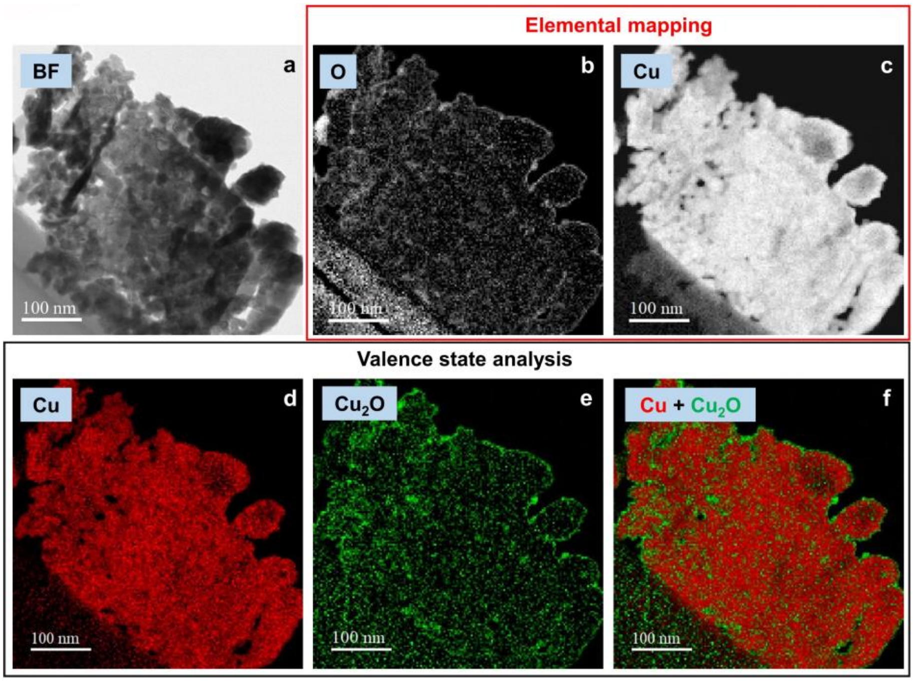

Supplementary Fig. 27 Elemental analysis of DVL-Cu after 50 h electrolysis in KCl. a STEM bright-field image of the 50 h post-electrolysis DVL-Cu in KCl. **b, c** EDS maps of Cu, O element in the 50 h post-electrolysis DVL-Cu in KCl. **d-f** EELS maps of Cu, Cu_2O and their overlay in the 50 h post-electrolysis DVL-Cu in KCl.

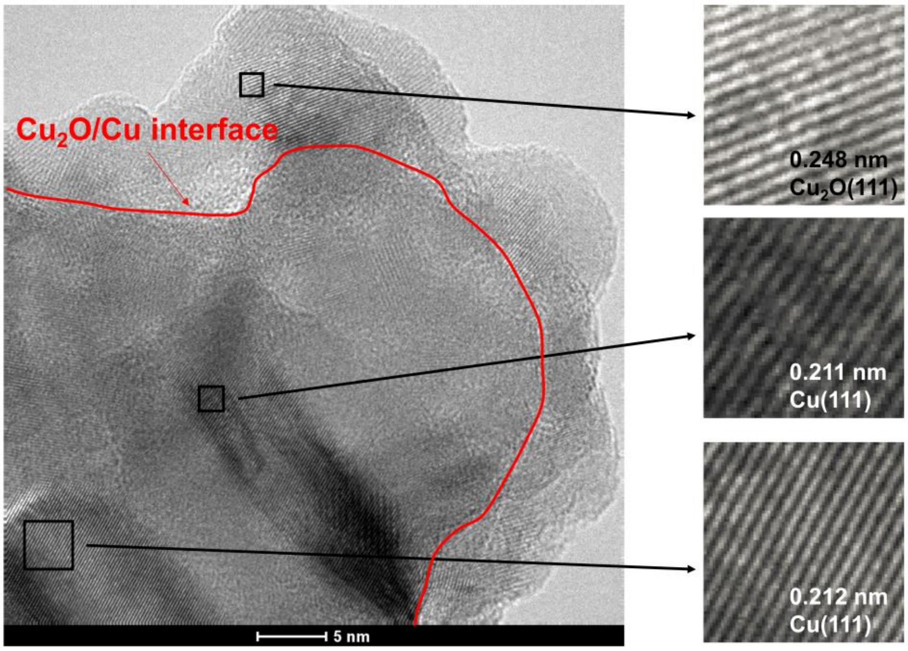

Supplementary Fig. 28 HRTEM image of DVL-Cu taken after 50 h CO_2RR. The DVL-Cu catalyst preserved a Cu/Cu_2O composite structure after the long-term test. The Cu_2O/Cu interface can be clearly seen in the image.

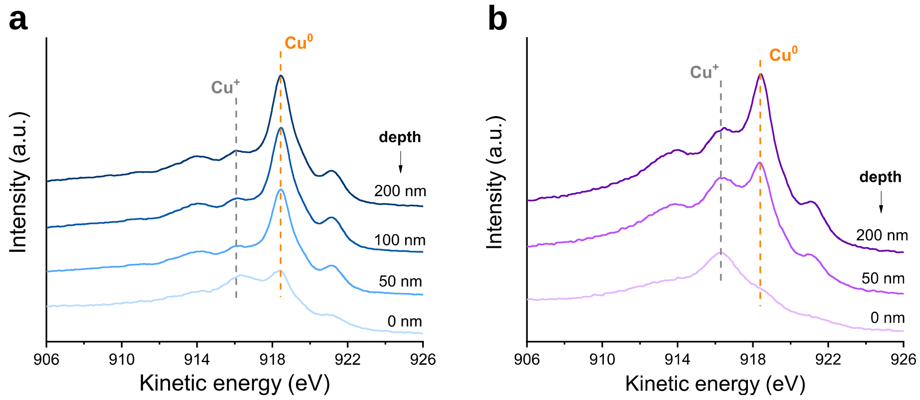

Supplementary Fig. 29 In-depth XPS analysis of post-electrolysis samples. a Cu *LMM* Auger spectra of the 20 h post-electrolysis DVL-Cu in $KHCO_3$ with respect to different Ar^+ etching depths. **b** Cu *LMM* Auger spectra of the 50 h post-electrolysis DVL-Cu in KCl with respect to different Ar^+ etching depths.

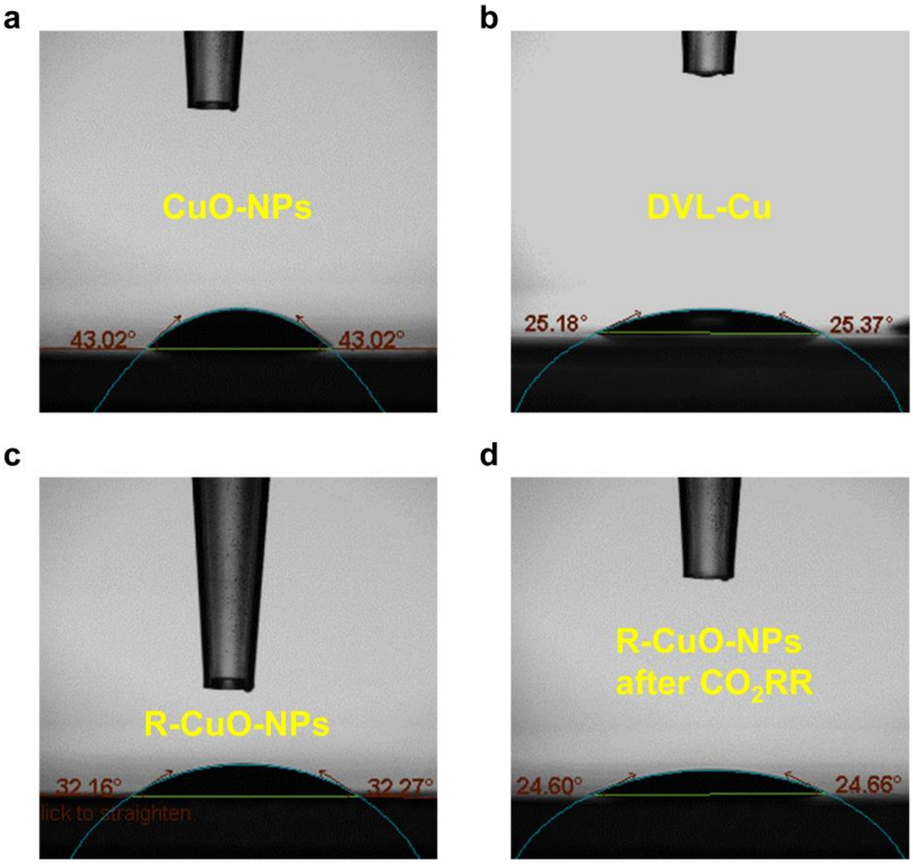

Supplementary Fig. 30 Water contact angle analysis of the samples. The water contact angles could serve as an indication of surface hydrophilicity. The contact angles of the samples before CO_2RR are larger than those after electrochemical reduction. The contact angles of DVL-Cu and R-CuO-NPs after CO_2RR are similar, suggesting similar hydrophilicity of the two samples during the catalysis process.

Supplementary Note 2: Electrolyte issue in CO_2RR industrialization.

As chloride ion (Cl^-) could discharge at the anode in KCl electrolyte, harmful to the device and environment. We did obsever that the Cl^- in catholyte would be transported through the membrane to anolyte when anion exchange membrane (AEM) was used in the flow cell system. Fortunately, although KCl as catholyte was used, the anolyte choice of H-cell system was arbitrary without difference in catalytic performance, and the Nafion-117 membrane can completely block Cl^- transmission. As shown in Supplementary Fig. 14, the Nafion membrane system display nearly the same FEs of ethylene and full-cell EEs at different current densities with the AEM system ($KHCO_3$ was chosen as the anolyte). Therefore, the catalytic performance of DVL-Cu was irrelevant with the anolyte and ion exchange membrane. Those anolytes with no Cl^- and membranes that could block the transportation of Cl^- would be suitable for industrialization in the future.

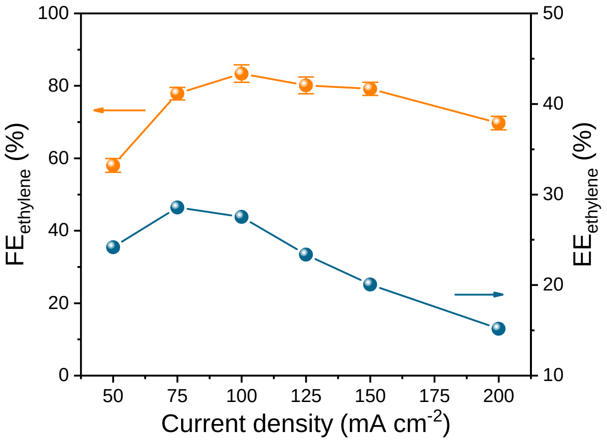

Supplementary Fig. 31 Ethylene FE and EE of DVL-Cu in flow cell using Nafion membrane. The Nafion membrane system display nearly the same FEs of ethylene and full-cell EEs at different current densities with the AEM system ($KHCO_3$ was chosen as the anolyte). Error bars represent the standard deviation of three independent measurements.

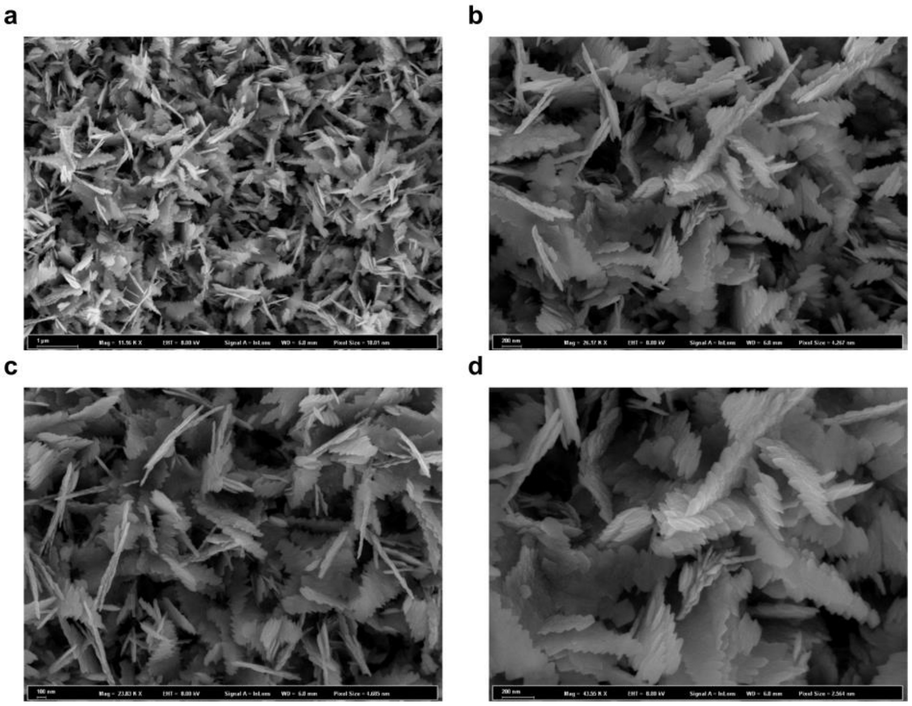

Supplementary Fig. 32 SEM images of CuO-NPs@GDL at different magnification. SEM images indicate that CuO-NPs@GDL has the same nanostructure as CuO-NPs.

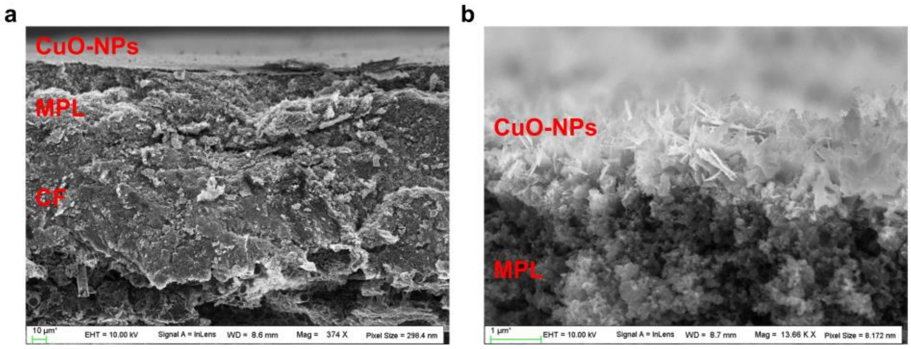

Supplementary Fig. 33 Cross-sectional SEM images of CuO-NPs@GDL. The CuO-NPs layer covers the surface of the microporous layer. The thickness of CuO-NPs is about 500 nm.

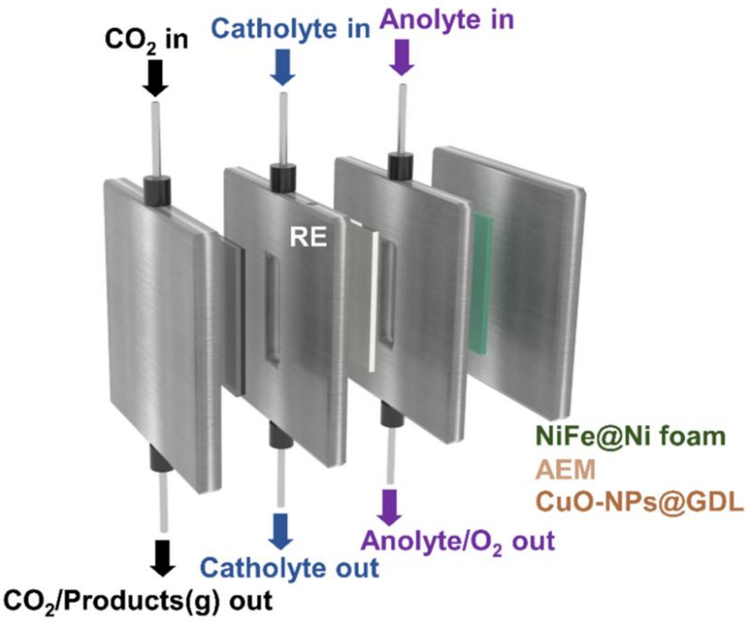

Supplementary Fig. 34 Schematic view of the flow cell used for CO_2RR test.

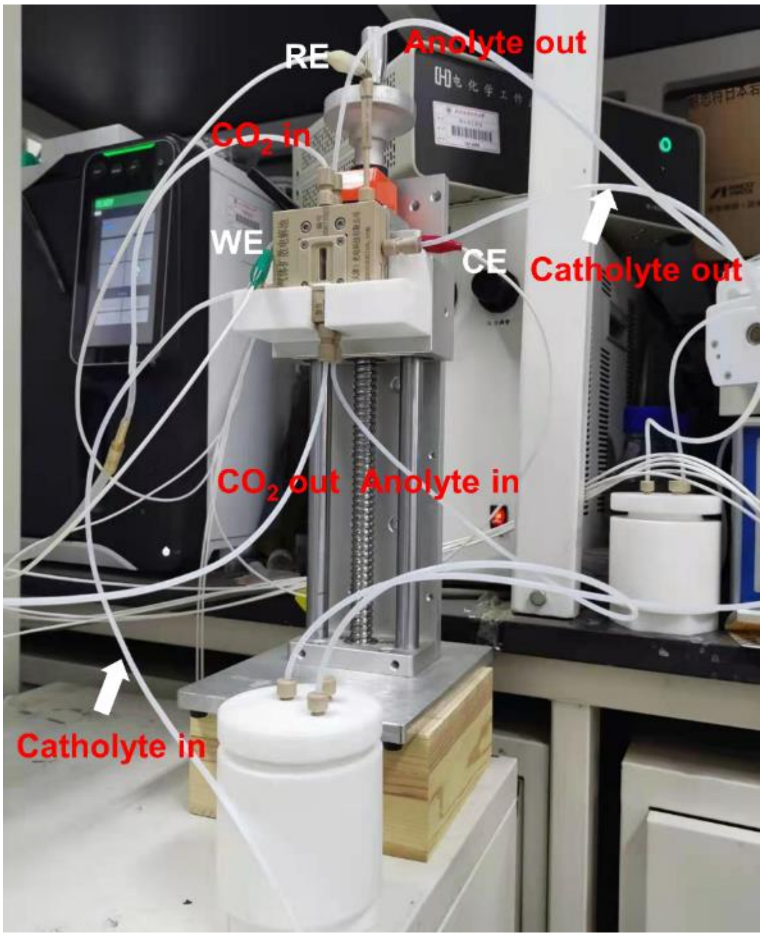

Supplementary Fig. 35 Optical image of the flow cell system used for CO_2RR test. The catholyte and anolyte were CO_2-saturated 0.5 M KCl and 2 M KOH solution, respectively. A peristaltic pump was employed to circulate the catholyte during the test, while a gas-liquid mixed-flow pump was used to circulate the anolyte. The CO_2 flow rate was controlled at 50 sccm through a mass flowmeter.

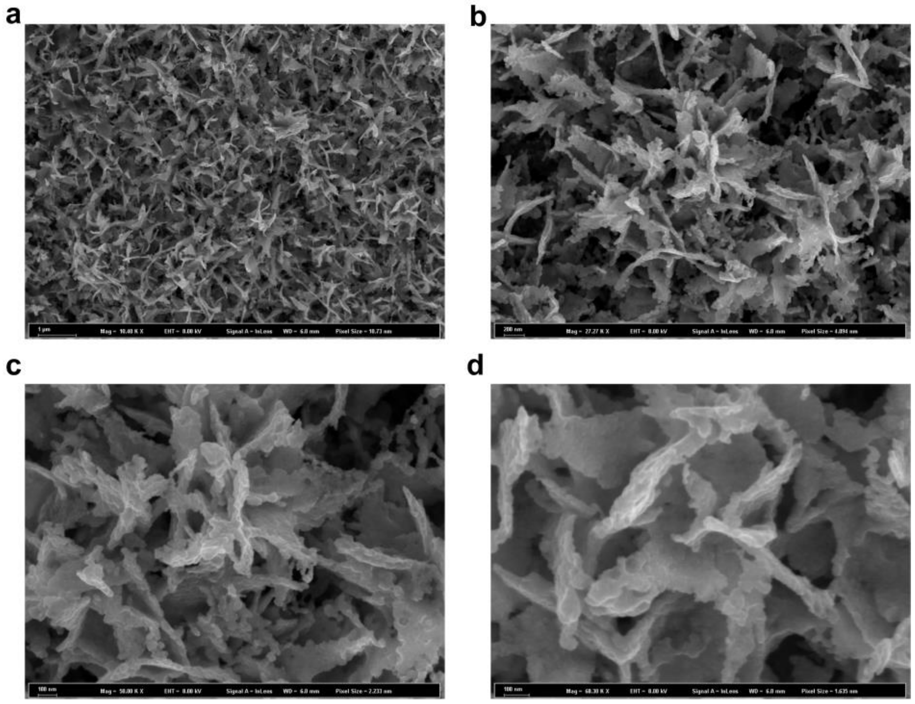

Supplementary Fig. 36 Different magnification SEM images of DVL-Cu@GDL. DVL-Cu@GDL retain the nanostructure of CuO-NPs. There is no agglomeration in DVL-Cu@GDL. The nanoplates are composed of nanoparticles rather than nanosheets.

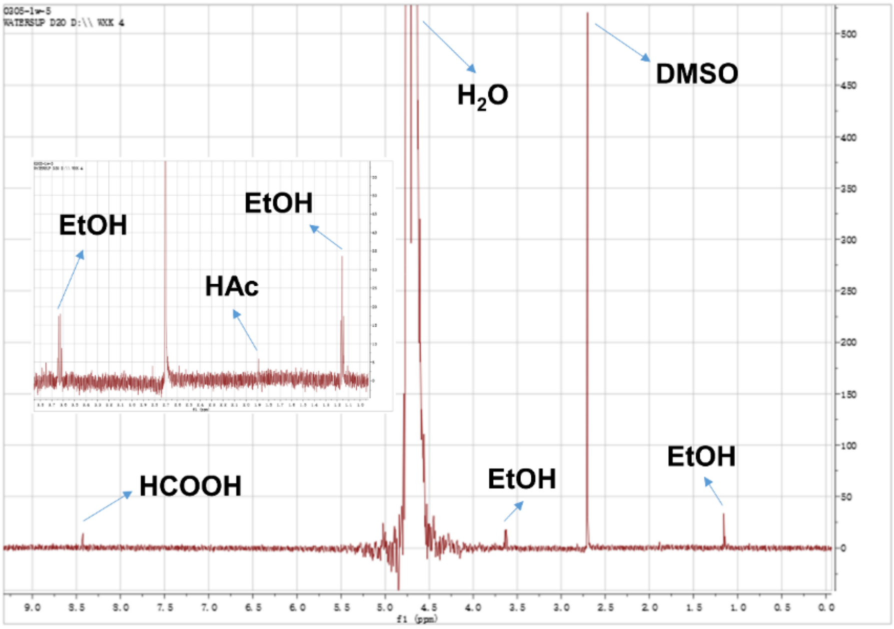

Supplementary Fig. 37 CO_2RR liquid products of the DVL-Cu@GDL catalyst. A representative ^{1}H-NMR spectrum of the electrolyte that collected after 1 h CO_2RR.

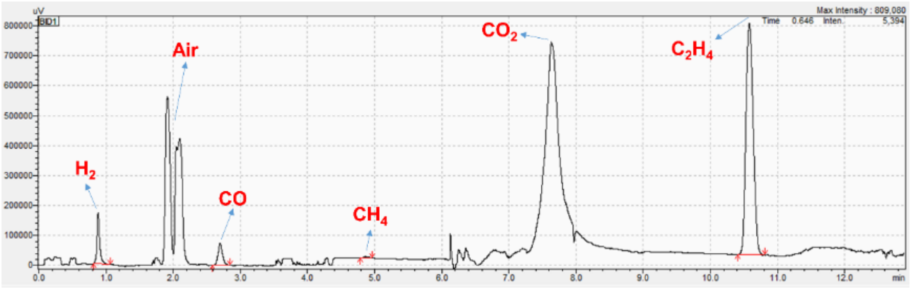

Supplementary Fig. 38 CO_2RR gas products of the DVL-Cu@GDL catalyst. The GC spectrum from the barrier ionization discharge (BID) detector at −0.81 V on DVL-Cu@GDL in the flow cell.

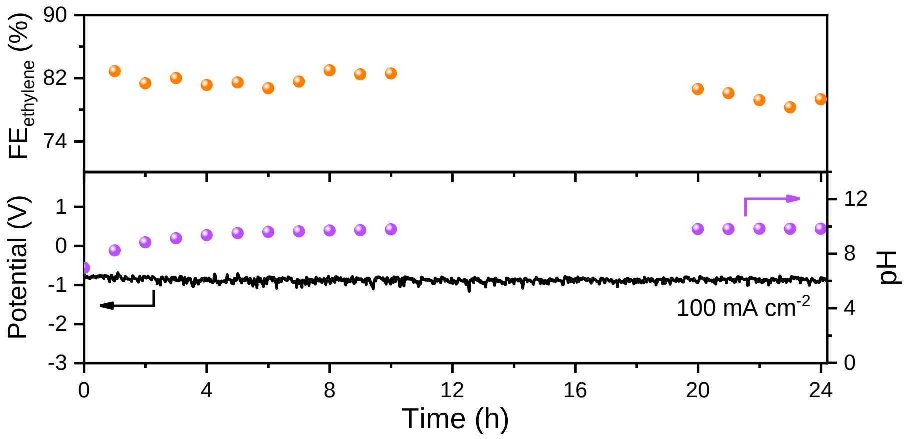

Supplementary Fig. 39 pH and ethylene FE during the long-term test in the flow cell. pH value retained around 9.8 in 6 h. pH value raised from 6.95 to 8.23 in the first 1h, and reached 9.50 after 5 h electrolysis. The pH value fluctuated around 9.8 for the rest of time. Despite pH changing over time, the FE of C_2H_4 remained stable around 80%, demonstrating that DVL-Cu delivers similar catalytic performance in the pH range from 6.5 to 9.8.

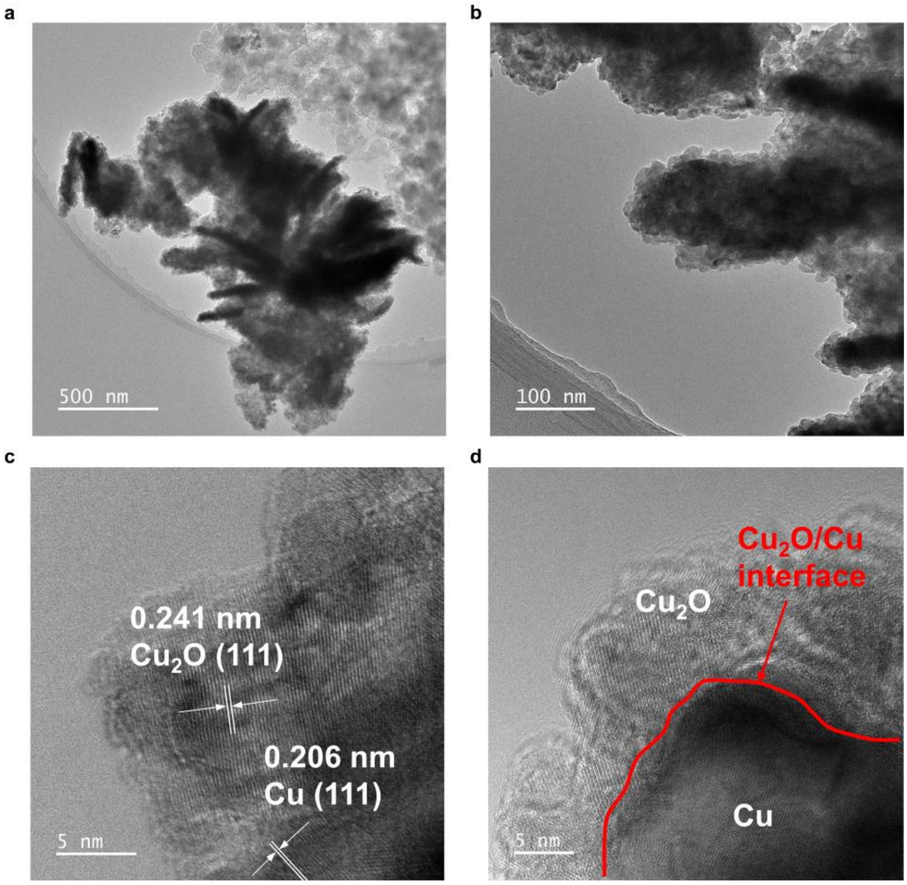

Supplementary Fig. 40 Different magnification TEM images of the DVL-Cu@GDL. The width of the DVL-Cu nanoplate is estimated to be about 500 nm, which is consistent with the thickness of the copper layer. The nanoplate is composed of smaller nanoparticles, which have Cu_2O shells and Cu cores. Cu_2O/Cu interfaces can be clearly seen in TEM images.

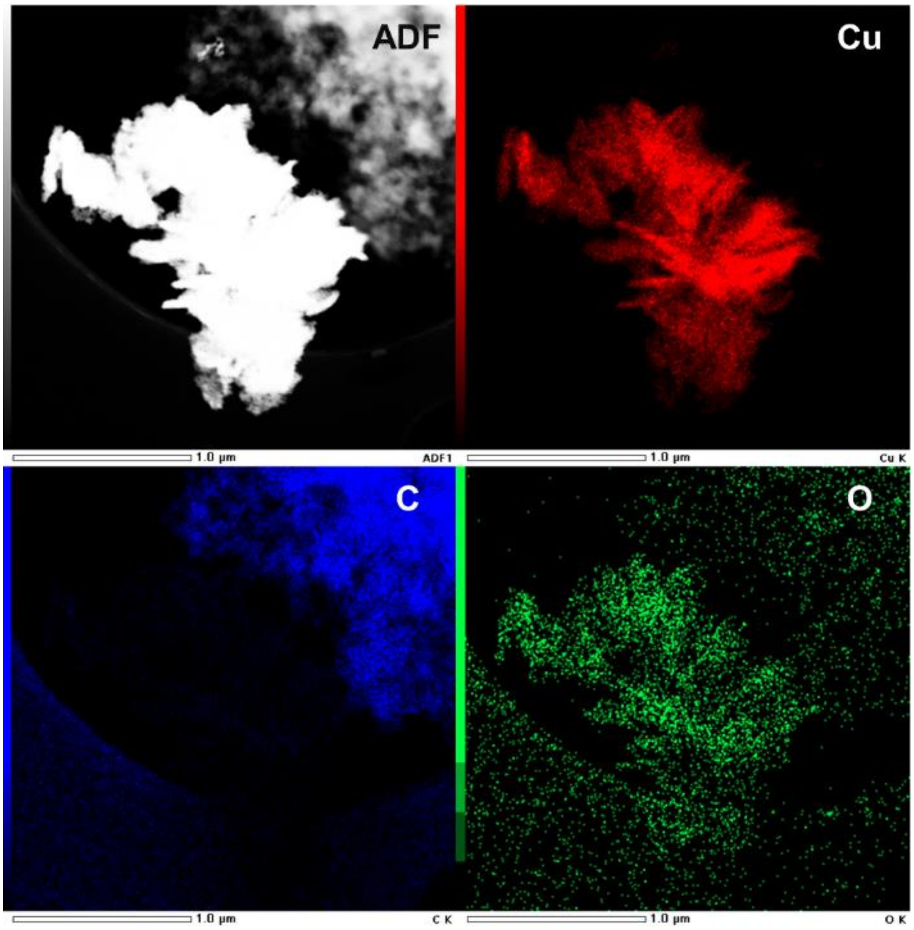

Supplementary Fig. 41 Annular-dark-field (ADF) image and EDS mapping of DVL-Cu@GDL. C elements come from the microporous layer and residual CO_2RR products. The overlapping distribution of Cu and O proves the presence of Cu_2O in DVL-Cu@GDL.

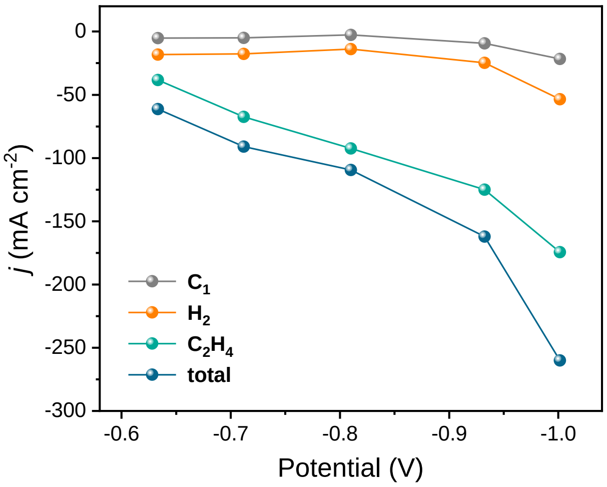

Supplementary Fig. 42 Total current density and partial current densities (C_2H_4, H_2, and C_1) of the DVL-Cu@GDL catalyst.

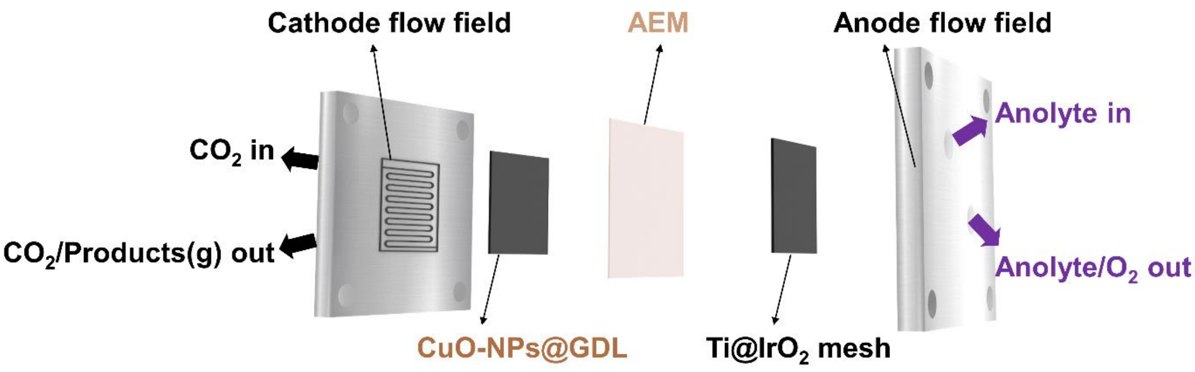

Supplementary Fig. 43 Schematic view of the MEA electrolyzer used for CO_2RR test.

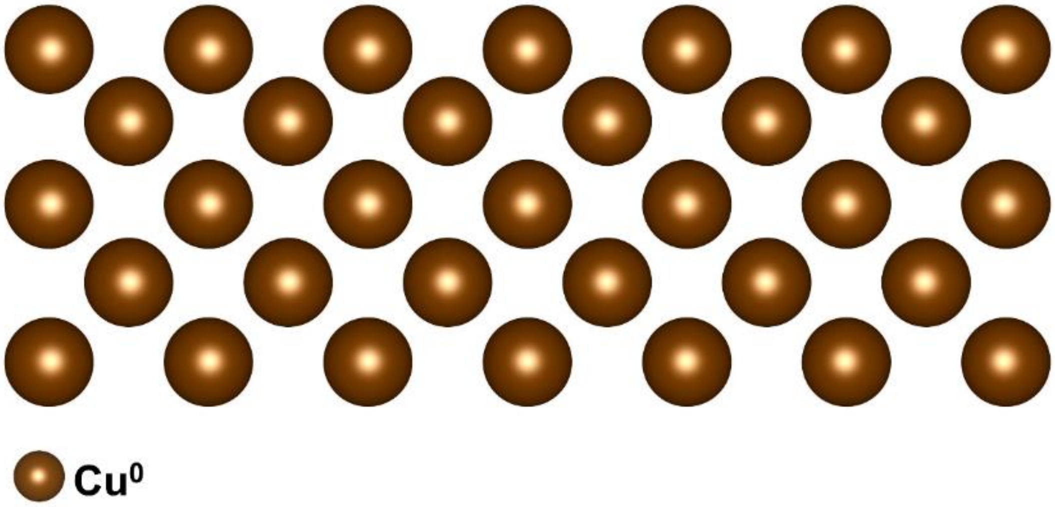

Supplementary Fig. 44 Geometry of Cu(110) slab for DFT calculations.

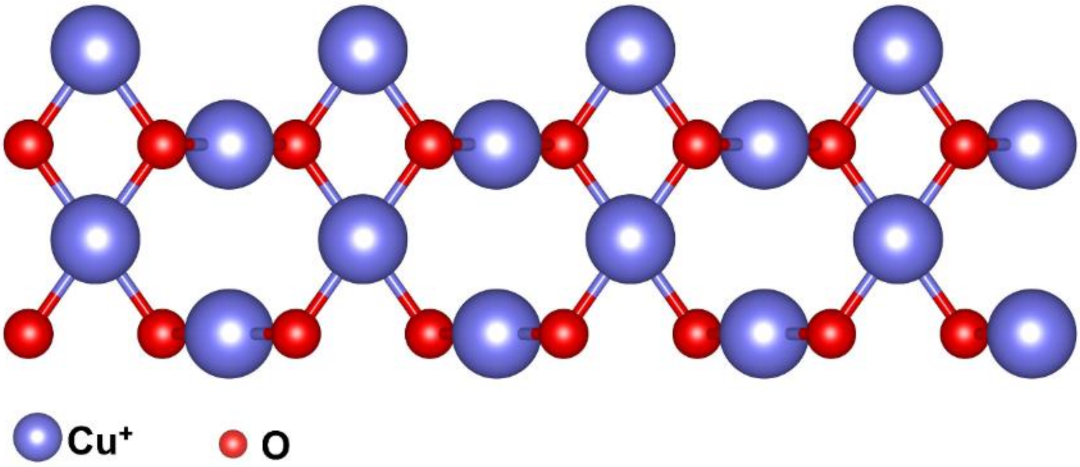

Supplementary Fig. 45 Geometry of $Cu_2O(110)$ slab for DFT calculations.

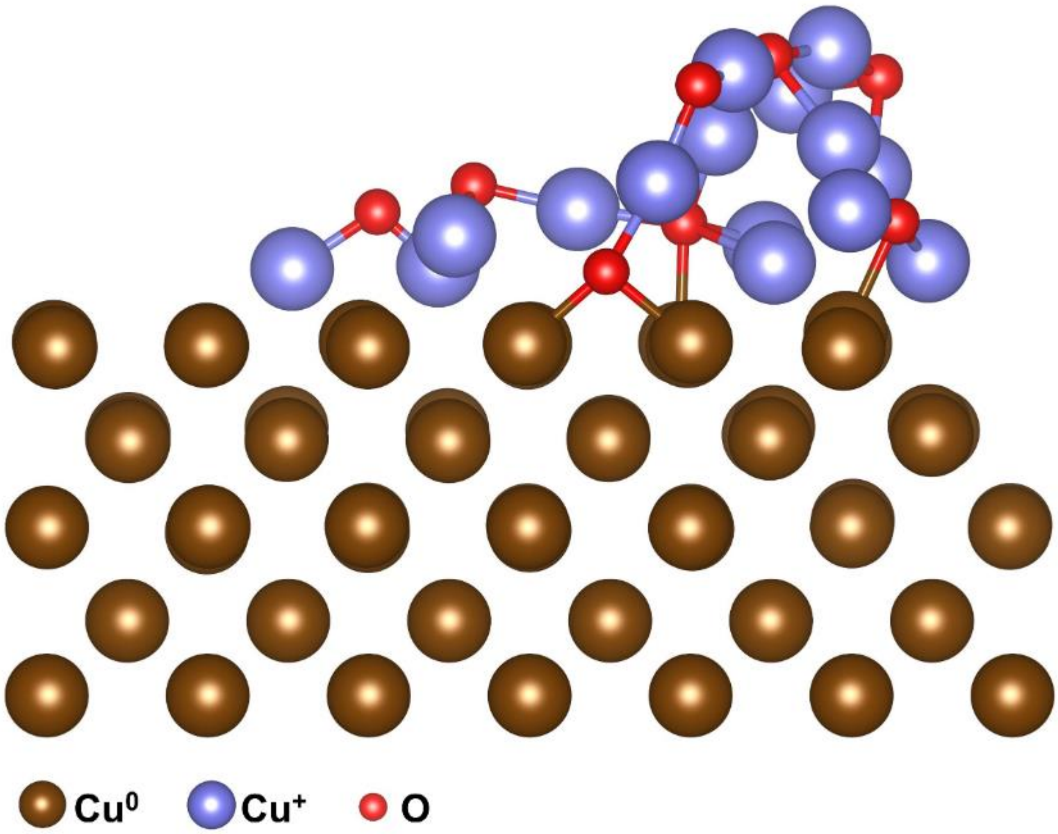

Supplementary Fig. 46 Geometry of Cu(110)/Cu_2O(110) configuration for DFT calculations.

Supplementary Note 3: Size effect of Cu_2O cluster to the DFT calculation results.

The binding strength of intermediates depends on the side of the Cu_2O cluster on the Cu surface, especially its size. Therefore, the composite Cu/Cu_2O structures with four different sized Cu_2O clusters, including 6 Å*8 Å with 5 Cu_2O, 10 Å*8 Å with 8 Cu_2O (the model used in the manuscript), 14 Å*8 Å with 12 Cu_2O, and 16 Å*8 Å with 16 Cu_2O (as shown in Supplementary Fig. 47b-e) was constructed. Refer to our previous DFT calculation results, the increased adsorption energy of the post-dimerization intermediate (*OCCOH) at Cu/Cu_2O interface reduce the energy barrier of C–C coupling step (the most important step of C_2 production), which is the essential origin of the superior catalytic performance of DVL-Cu. Meanwhile, the *CO+*CO→*CO+*COH is the rate-determining step (RDS) of ethylene production at the Cu/Cu_2O interface and Cu(110) slab. Hence, we calculated the free energy of the following reaction steps (as shown in Supplementary Fig. 47a): *CO→*OCCOH. DFT calculation results reveal that the free energies of C-C coupling steps are exergonic on all Cu/Cu_2O models of different cluster sizes, indicating that the cluster sizes have no impact on the increased adsorption capacity of the post-dimerization intermediate at the Cu/Cu_2O interfaces. Moreover, the energy barriers of *CO+*CO hydrogenation remain moderate (0.59-0.66 eV) on all Cu/Cu_2O models, lower than that on the Cu(110) slab (1.15 eV) and Cu_2O(110) slab (0.75 eV) and the RDS of other competing by-products, which are consistent with our DFT conclusions. Furthermore, as the C_2H_4 desorption is the RDS of ethylene production on Cu_2O(110) and is exergonic only on Cu/Cu_2O interfaces (Cu_2O size: 10 Å*8 Å) according to the previous calculation results, the reaction free energies of C_2H_4 desorption were also calculated. As shown in Supplementary Table 1, the reaction free energies are exergonic on Cu/Cu_2O interfaces of different Cu_2O cluster sizes, indicating that the Cu/Cu_2O interfaces of different Cu_2O cluster sizes possess a fast C_2H_4 production capacity equally. These results suggest that the DFT conclusions remain same and agree with the experimental trend when altering the size of adsorbed Cu_2O clusters.

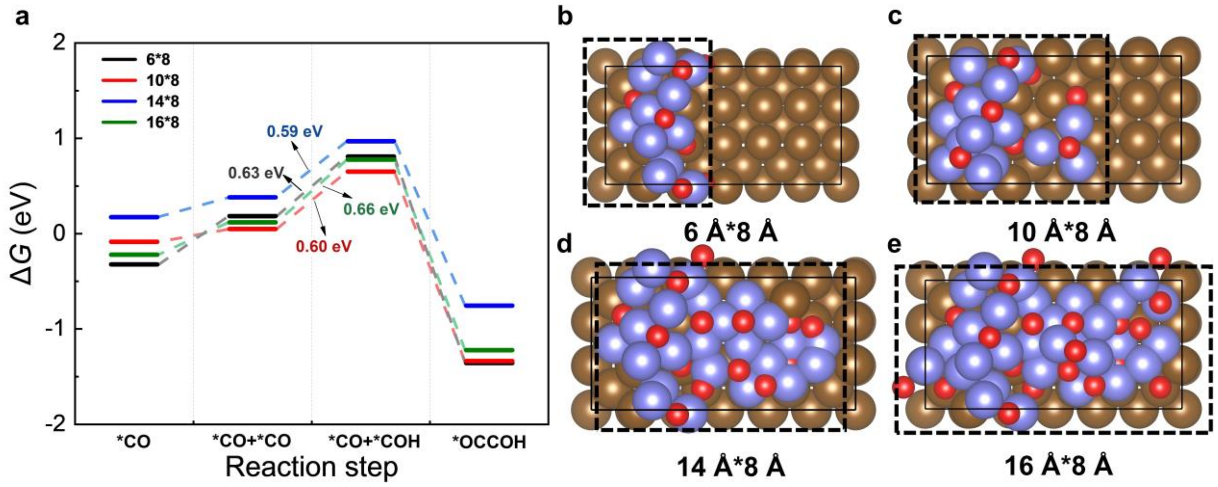

Supplementary Fig. 47 Size effect of Cu_2O cluster. a A reaction energy diagram for *CO→*OCCOH on the Cu/Cu_2O interface of different Cu_2O cluster sizes. **b-e** Geometry of Cu(110)/Cu_2O(110) configurations of different Cu_2O cluster sizes for DFT calculations. The sizes marked in the **b-e** are for Cu_2O.

Supplementary Table 1. Reaction free energy of C_2H_4 desorption on different reaction sites.

Reaction sites	Reaction free energy of C_2H_4 desorption
Cu(110)	0.46 eV
Cu_2O(110)	1.19 eV
Cu/Cu_2O (6 Å*8 Å)	−0.44 eV
Cu/Cu_2O (10 Å*8 Å)	−0.52 eV
Cu/Cu_2O (14 Å*8 Å)	−0.49 eV
Cu/Cu_2O (16 Å*8 Å)	−0.57 eV

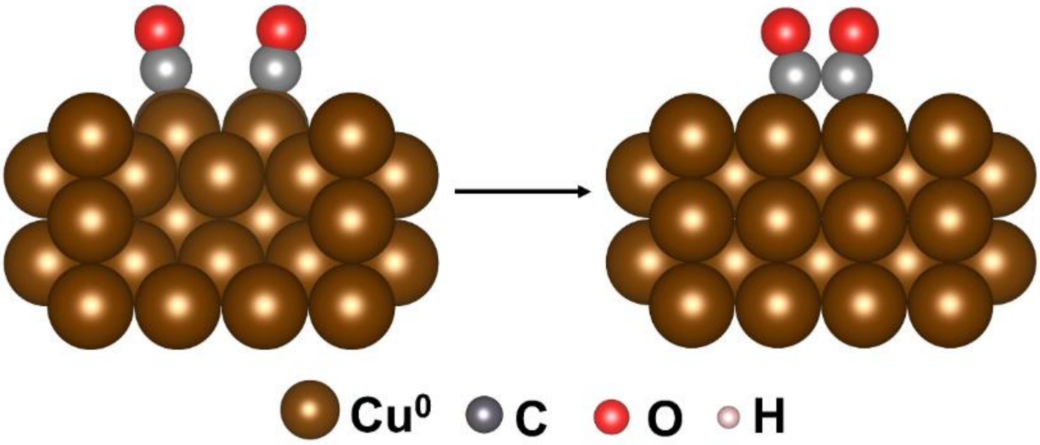

Supplementary Fig. 48 Optimized adsorption structures before and after *CO/*CO dimerization on Cu(110) sites. The dimerization of two *CO (*CO+CO→*OCCO) is kinetics forbidden on Cu(110) sites as the *OCCO would divide into two *CO spontaneously after dimerization.

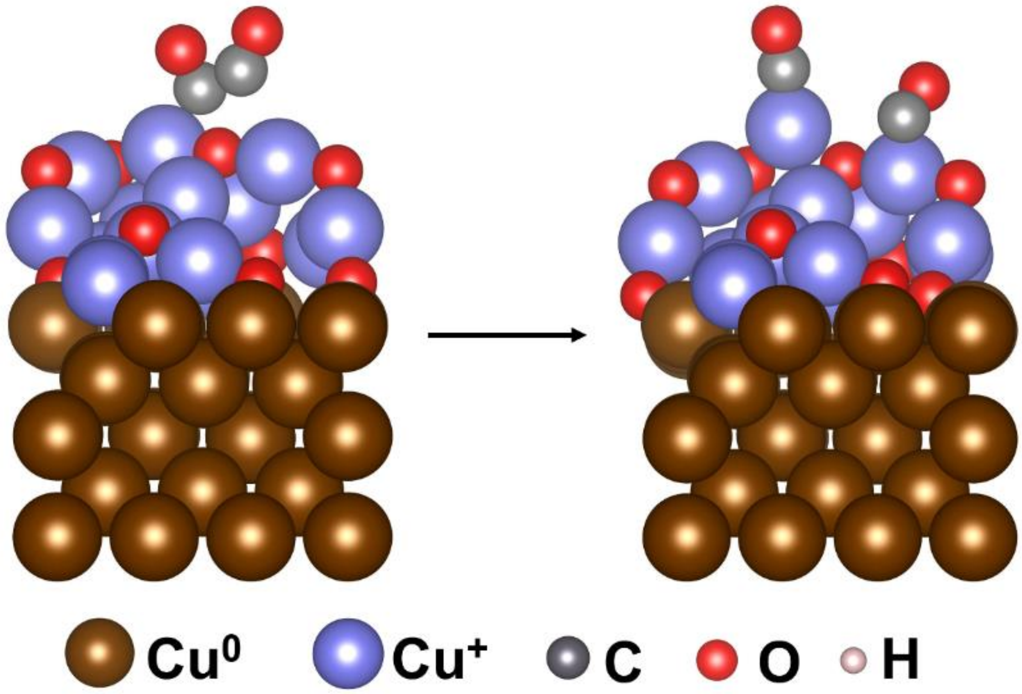

Supplementary Fig. 49 Optimized adsorption structures before and after *CO/*CO dimerization on Cu_2O (110) sites. The dimerization of two *CO (*CO+CO→*OCCO) is kinetics forbidden on Cu_2O(110) sites as the *OCCO would divide into two *CO spontaneously after dimerization.

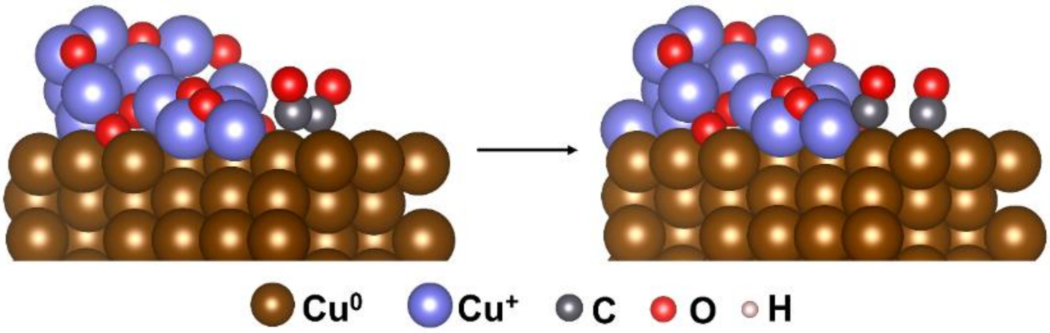

Supplementary Fig. 50 Optimized adsorption structures before and after *CO/*CO dimerization on Cu(110)/Cu_2O(110) interface. The dimerization of two *CO (*CO+CO→ *OCCO) is kinetics forbidden on Cu(110)/Cu_2O(110) interface as the *OCCO would divide into two *CO spontaneously after dimerization.

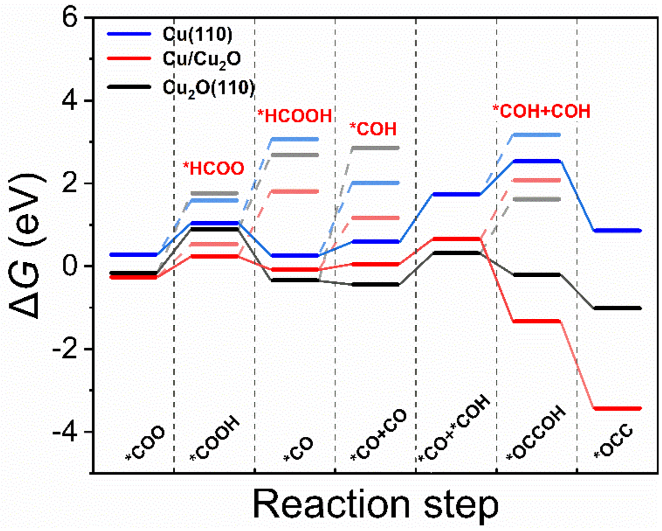

Supplementary Fig. 51 A reaction energy diagram for CO_2RR on Cu(110) slab, Cu/Cu_2O interface and Cu_2O(110) slab. Intermediates corresponding to lighter colors are marked in red font.

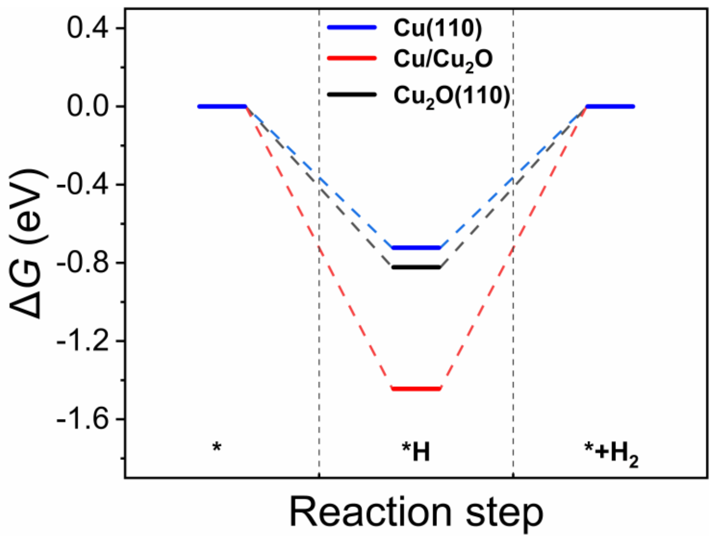

Supplementary Fig. 52 A reaction energy diagram for HER on Cu(110) slab, Cu/Cu_2O interface and Cu_2O(110) slab. Intermediates corresponding to lighter colors are marked in red font.

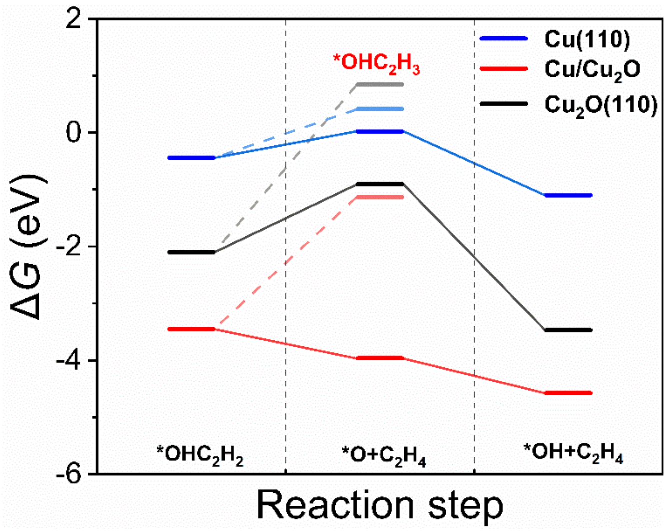

Supplementary Fig. 53 A reaction energy diagram for CO_2RR to ethylene and ethanol on Cu(110) slab, Cu/Cu_2O interface and Cu_2O(110) slab.

Supplementary Table 2 Comparison of reported Cu-based CO_2RR catalysts toward C_2H_4 production

Catalysts	Electrolyte	Potential (V vs. RHE)	$FE_{C_2H_4}$ (%)	$j_{C_2H_4}$ (mA cm^{-2})	Maximum $EE_{C_2H_4}$ (%)*	Reference
DVL-Cu	0.5 M KCl	−0.81	84.5	92.5	28.9	This work
		−1.01	67.1	174.4		
OBC	0.5 M $KHCO_3$	−1.00	45	44.7	/	2
O_2-plasma-treated Cu	0.1 M $KHCO_3$	−0.9	60	7.2	/	3
Cu 3D CTPI	/	/	69	304	22	4
Cu(B)-2	0.1 M KCl	−1.1	52	36.4	/	5
Cu-12	1 M $KHCO_3$	−0.83	72	230	20	6

*The maximum $EE_{C_2H_4}$ was not achieved in the potentials listed in the table.

Supplementary Table 3 FEs of DVL-Cu at different potentials in the H-cell.

Potential (V)	FE (%)									
	C_2H_4	H_2	CO	CH_4	formate	ethonal	acetate	Pr-OH	C_2H_6	Total
**−0.6**	43.8	39.7	10.8	0.0	1.6	1.5	0.1	0.5	0.0	97.9
**−0.7**	63.9	28.1	1.4	0.7	0.6	3.0	0.1	0.4	0.0	98.1
**−0.8**	69.8	20.4	2.6	0.3	0.9	2.8	0.1	0.8	0.8	98.5
**−0.9**	74.9	13.6	2.9	0.8	1.0	3.3	0.1	0.8	1.4	98.9
**−1.0**	66.3	17.2	2.6	4.8	0.7	4.8	0.1	0.8	1.9	99.2
**−1.1**	60.8	22.3	1.5	5.9	1.4	5.5	0.0	0.4	0.5	98.3

Supplementary Table 4 Contributions to the free energy from the zero-point energy correction (E_{ZPE}), enthalpic temperature correction (ΔH_T), gas correction (ΔH_G), entropy (TS), and the calculated total free energy, respectively. The literature entropies taken from NIST are also listed. All values are given in eV.

Gas phase	E_{DFT}	E_{ZPE}	TS	ΔH_T	ΔH_G	G
CO_2 (g)	−22.95	0.31	0.67	0.10	0.13	−23.08
CO (g)	−14.78	0.13	0.60	0.09	−0.51	−15.66
H_2 (g)	−6.83	0.27	0.41	0.09	−0.06	−6.95
H_2O (g)	−14.22	0.57	0.67	0.10	−0.08	−14.28
C_2H_4 (g)	−31.98	0.89	0.68	0.13	−0.22	−31.86

Supplementary Table 5 Calculated free energy of each CO_2RR intermediate at 0 V (vs. RHE) and all values are given in eV.

Species	G (eV)		
	Cu(110)	Cu/Cu_2O	Cu_2O(110)
*	0.000	0.000	0.000
*CO_2	0.279	−0.264	−0.161
*COOH	1.036	0.237	0.894
*CO	0.257	−0.085	−0.344
*CO+*CO	0.587	0.048	−0.442
*CO+*CHO	1.736	0.652	0.311
*OCCOH	2.528	−1.337	−0.210
*OCC	0.857	−3.432	−1.018
*OCCH	0.475	−2.733	−0.729
*OHCCH	0.233	−2.886	−2.237
*OH_2C_2H	−0.440	−3.450	−2.099
*O	0.023	−3.965	−0.907
*OH	−1.096	−4.579	−3.471

Supplementary Reference

1. Verdaguer-Casadevall, A. et al. Probing the active surface sites for CO reduction on oxide-derived electrocatalysts. *J. Am. Chem. Soc.* **137**, 9808–9811 (2015).
2. Zhang, W. et al. Atypical oxygen-bearing copper boosts ethylene selectivity toward electrocatalytic CO_2 reduction. *J. Am. Chem. Soc.* **142**, 11417-11427 (2020).
3. Mistry, H. et al. Highly selective plasma-activated copper catalysts for carbon dioxide reduction to ethylene. *Nat. Commun.* **7**, 12123 (2016).
4. Ozden, A. et al. High-Rate and Efficient Ethylene Electrosynthesis Using a Catalyst/Promoter/Transport Layer. *ACS Energy Lett.* **5**, 2811-2818 (2020).
5. Zhou, Y. et al. Dopant-induced electron localization drives CO_2 reduction to C_2 hydrocarbons. *Nat. Chem.* **10**, 974-980 (2018).
6. Li, F. et al. Molecular tuning of CO_2-to-ethylene conversion. *Nature* **577**, 509-513 (2020).
